# FOXL2 interaction with different binding partners regulates the dynamics of ovarian development

**DOI:** 10.1126/sciadv.adl0788

**Published:** 2024-03-22

**Authors:** Roberta Migale, Michelle Neumann, Richard Mitter, Mahmoud-Reza Rafiee, Sophie Wood, Jessica Olsen, Robin Lovell-Badge

**Affiliations:** ^1^ Laboratory of Stem Cell Biology and Developmental Genetics, The Francis Crick Institute, London NW1 1AT, UK.; ^2^ Bioinformatics core, The Francis Crick Institute, 1 Midland Road, London NW1 1AT, UK.; ^3^ RNA Networks Laboratory, The Francis Crick Institute, 1 Midland Road, London NW1 1AT, UK.; ^4^ Genetic Modification Service, The Francis Crick Institute, 1 Midland Road, London NW1 1AT, UK.

## Abstract

The transcription factor FOXL2 is required in ovarian somatic cells for female fertility. Differential timing of *Foxl2* deletion, in embryonic versus adult mouse ovary, leads to distinctive outcomes, suggesting different roles across development. Here, we comprehensively investigated FOXL2’s role through a multi-omics approach to characterize gene expression dynamics and chromatin accessibility changes, coupled with genome-wide identification of FOXL2 targets and on-chromatin interacting partners in somatic cells across ovarian development. We found that FOXL2 regulates more targets postnatally, through interaction with factors regulating primordial follicle formation and steroidogenesis. Deletion of one interactor, ubiquitin-specific protease 7 (*Usp7*), results in impairment of somatic cell differentiation, germ cell nest breakdown, and ovarian development, leading to sterility. Our datasets constitute a comprehensive resource for exploration of the molecular mechanisms of ovarian development and causes of female infertility.

## Teaser

Chromatin proteomics of FOXL2 interactome across ovary development uncovers a role for USP7 in granulosa cell differentiation.

## INTRODUCTION

The gonads develop from bipotential genital ridges, which contain undifferentiated cell types poised to develop into ovaries in females or testes in males (*1*). The presence or absence of the Y-linked testis determination gene *Sry* determines which type of gonad it will become (*2*). Its expression is sufficient to induce testis differentiation in an XY or XX context (*3*, *4*). This is also the case for its downstream target *Sox9* (*5*).

Ovarian development was considered as a default pathway activated in the absence of *Sry*, but this notion was challenged by data showing that sex determination is an active process, being governed by antagonistic signals that promote the male or female pathway and silence the alternative one (*6*–*8*). However, the ovarian pathway does not seem to have a clear-cut hierarchy, operating instead as a complex network of genes with considerable redundancy. *Foxl2*, a gene encoding for the forkhead box L2 (FOXL2) transcription factor (TF) is one such critical player and may have multiple roles in ovary morphogenesis, being expressed in the supporting cells of the ovary, namely, granulosa cells (*9*, *10*).

Previous work has highlighted the importance of FOXL2 in many aspects of female sexual development (*11*) as well as in granulosa cell–related pathologies (*12*). Heterozygous mutations in the *FOXL2* gene in humans have been linked to premature ovarian insufficiency (POI) and infertility as part of the Blepharophimosis, ptosis, and epicanthus inversus syndrome (*13*–*15*). Murine models have helped to pinpoint the role of FOXL2 in this syndrome. *Foxl2^−/−^* ovaries become dysgenic from 1 week postpartum, and granulosa cells fail to differentiate and to undergo the squamous-to-cuboidal transition typical of primary follicles, resulting in infertility. This is due to the inability of granulosa cells lacking FOXL2 to undergo proper differentiation, resulting in impaired folliculogenesis. The lack of secondary follicles in this model also suggested an indirect role of FOXL2 in follicle activation exerted by controlling genes directly involved in this process within granulosa cells, namely, *Cdkn1b/p27* (*16*), S*mad3* (*17*), and *Kitl* (*18*), as well as by regulating the growth of the oocytes, which, in turn, has an effect on primordial follicle activation (PFA) (*10*). Mutant ovaries showed not only down-regulation of somatic sex markers of folliculogenesis but also up-regulation of the pro-testis factor *Sox9* as well as others male gonadal-specific markers including *Fgf9*, *Dmrt1*, and *Gata4* (*19*, *20*). However, this only happens postnatally, indicating that FOXL2 is not critical for embryonic ovary specification but essential for granulosa cell fate maintenance.

In contrast, the conditional deletion of *Foxl2* from adult ovaries resulted in rapid transdifferentiation of granulosa cells into Sertoli cells, their opposite sex counterpart (*7*). This study demonstrated the plasticity of the adult ovary and the crucial role played by FOXL2 in ovarian sex maintenance. More recently, a similar role was discovered for TRIM28, which we showed to be recruited on chromatin together with FOXL2 (*6*). Collectively these data support a differential role played by FOXL2 depending on the developmental stage, as well as suggesting the presence of additional factors cooperating with FOXL2 in ensuring correct ovarian development and function.

We postulate that the multiple roles of FOXL2 during development may be dependent on the availability of different interacting partners. To address this, we performed a chromatin immunoprecipitation combined with selective isolation of chromatin-associated Proteins (ChIP–SICAP) analysis to identify proteins that localize with FOXL2 on chromatin and genome-wide target genes and then integrated these with RNA sequencing (RNA-seq) and assay for transposase-accessible chromatin with high-throughput sequencing (ATAC-seq) data on FOXL2 expressing cells purified from a *Foxl2^EGFP^* mouse line across ovarian development. Our data indicate a more prominent role of FOXL2 in postnatal stages. Our integrative approach uncovered an interactor with a role in folliculogenesis and gonadal development: ubiquitin-specific protease 7 (USP7). We show that USP7 is necessary for proper folliculogenesis, as conditional deletion in somatic cells of the ovary leads to defects in germ cell nest breakdown in the pre-pubertal ovary, absence of follicles beyond the primordial stage, and complete ovarian degeneration in the adult.

## RESULTS

### ChIP-SICAP reveals differential genomic binding of FOXL2 throughout ovarian development

**Fig. 1.**
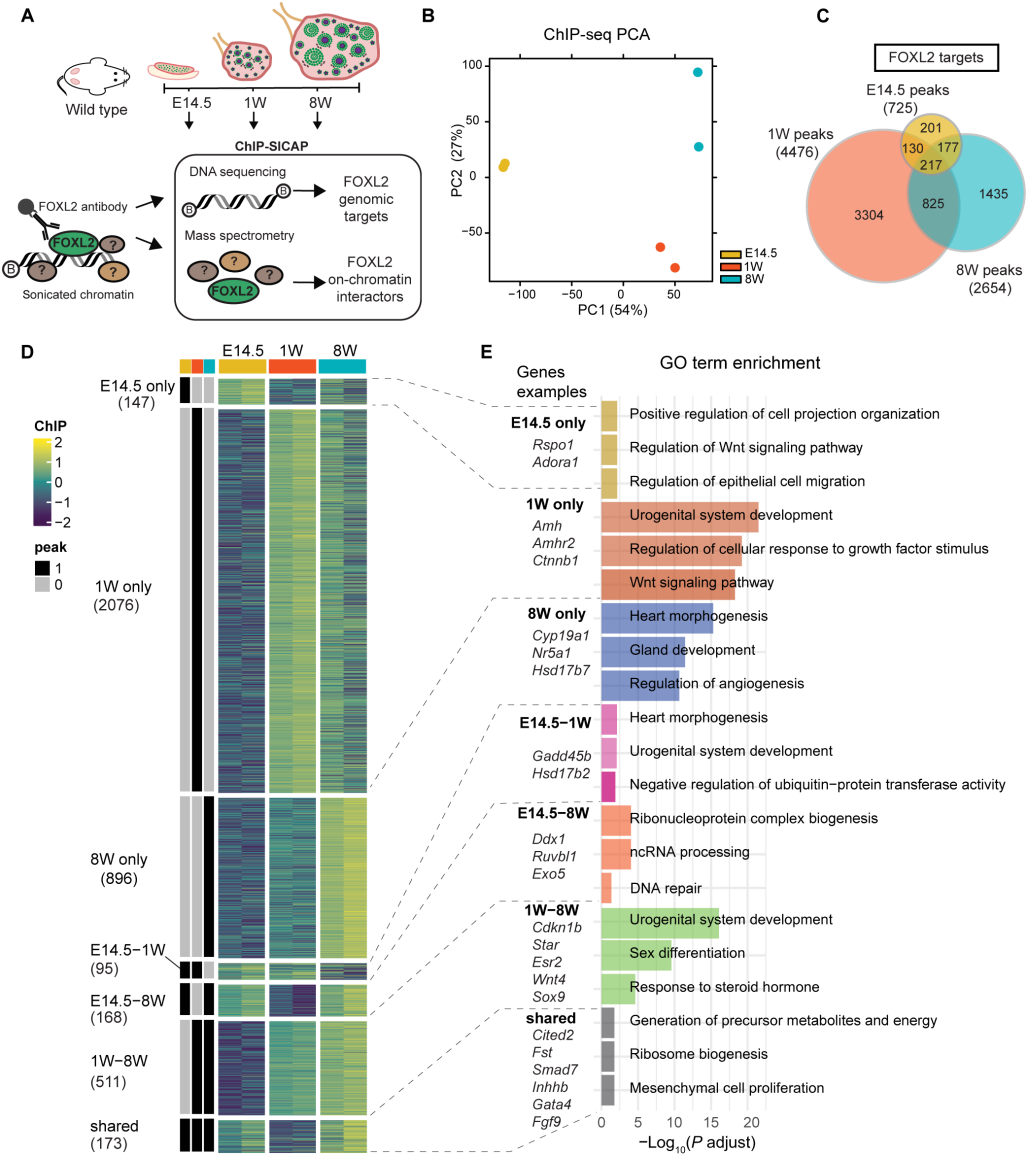
ChIP-SICAP time course reveals differential FOXL2 DNA binding across ovarian development. (**A**) Experimental design of FOXL2 ChIP–SICAP performed on mouse ovaries collected at E14.5, 1W, and 8W, *n* = 2 biological replicates. A FOXL2-specific antibody was used to capture genomics fragments bound by FOXL2 as well as proteins colocalizing with FOXL2 on chromatin. (**B**) Principal components analysis (PCA) of FOXL2 ChIP-seq consensus peaks. (**C**) Venn diagram depicting the number of peaks identified at each time point and their overlap. (**D**) ChIP peak occupancy scores (gene level). Heatmap splitting is based on ChIP peak presence/absence as indicated by black/gray bars (left). Rows are scaled by *z*-score. The number of genes per cluster is shown below the cluster name. (**E**) Bar chart showing representative GO biological processes (BPs).

To profile the dynamic changes in FOXL2 DNA binding and to identify its on-chromatin protein interactors, we performed a modified version of ChIP-SICAP [Materials and Methods and (*21*, *22*)] on whole mouse ovaries at three time points: E14.5, 1 week (1W) and 8 weeks (8W) postnatally (Fig. 1A). ChIP-SICAP is a dual approach that allowed to simultaneously identify genome-wide FOXL2 binding sites, via ChIP sequencing (ChIP-seq), and proteins colocalizing with FOXL2 on chromatin, via mass spectrometry. Antibody specificity was validated by immunofluorescence on tissue sections of wild-type adult ovaries, ovaries where *Foxl2* was conditionally deleted as previously shown (*7*), and on wild-type testis sections (fig. S1A). E14.5 was chosen because this embryonic stage is characterized by high FOXL2 expression in the pre-granulosa cells of the ovarian medulla (*23*); 1W coincides with the activation of primordial follicles into primary follicles and corresponds to the time point at which SOX9 protein is first detected in *Foxl2^−/−^* ovaries (*10*); at 8W, FOXL2 is widely expressed in the follicles where it regulates folliculogenesis (*11*) and maintains ovarian cell fate (*7*). Principal components analysis (PCA) of ChIP-seq peaks highlights the differences between FOXL2 binding sites across the time course (Fig. 1B). First principal component 1 (PC1) showed a clear separation of the E14.5 samples from both 1W and 8W. PC2 further separated the 1W and 8W samples. Peak annotation showed that embryonic and postnatal FOXL2 binding sites differed in their genomic location: Most identified at E14.5 (50%) were mapped to promoters, while only about 30% of peaks identified at 1W and 8W were located within promoters (fig. S1B). Motif enrichment analysis of consensus peaks confirmed enrichment of FOXL2 and other forkhead factors motifs (fig. S1C). Furthermore, we found enrichment of steroid hormone receptors binding sites among the top 10 predicted motifs. These included those for estrogen and androgen receptors (ESR1, *ESRRB*, AR), known to regulate hormone production and granulosa cell proliferation throughout ovarian development (*24*, *25*), as well as steroidogenic factor 1 (NR5A1), a master regulator of many aspects of gonadal development including steroidogenesis (*26*), previously identified as a FOXL2 binding partner in vitro (*27*) and essential for gonadal development in both sexes (*28*).

In total, we detected 725 peaks at E14.5, 4476 at 1W, and 2654 at 8W (Fig. 1C and data files S1 and S2). Among all FOXL2 targets, we identified known ones bound throughout the time course, such as *Cited2* (fig. S1D) (*29*), as well as targets dynamically bound including *Esr2*. Moreover, other potential target genes were uncovered, such as *Rspo1*, a positive effector of β-catenin signaling required for ovarian development (*30*). These targets, in addition to other known FOXL2 targets including *Fst*, *Cdkn1b*, *Fgf9*, and enhancers of *Sox9*, displayed a similar profile of FOXL2 binding as identified by a previously published study focused on E14.5 mouse ovaries, assessed by standard ChIP-seq (fig. S2) (*29*). Of note, we did not detect binding events to some of the genes identified in this study, for example, *Dmrt1*, *Cited1*, or *Erbb4*.

Pairwise differential binding analysis allowed the identification of FOXL2-binding events that were statistically different between time points (data file S1). We detected the greatest number of differential binding (2503 differential peaks) when comparing E14.5 with 8W, followed by the comparison between E14.5 and 1W (2098 differential peaks), and lastly by the comparison between 1W and 8W (634 differential peaks). By integrating our FOXL2 ChIP-seq dataset with a microarray analysis of genes differentially expressed at 13.5 days postcoitum (dpc), at 16.5 dpc, and at birth in wild-type controls versus mouse ovaries lacking *Foxl2* [*Foxl2*^−/−^; (*18*); data file S2], we aimed to discriminate between activation and repression activity of FOXL2 on the targets that we identified. A total of 770 genes were classified as more likely to be activated by FOXL2, because they were down-regulated in the absence of *Foxl2* as well as bound by FOXL2 (fig. S3A).

Besides *Cited2* and *Esr2*, this group included the cell-cycle repressor gene *Cdkn1b* (fig. S3B), a known FOXL2 target that regulates granulosa cell proliferation (*16*); *Smad7* that encodes for a regulator of PFA together with SMAD3 (*17*) (fig. S2C); and the follistatin gene (*Fst*) that encodes a secreted inhibitor of the TGFβ pathway, essential for ovarian development (fig. S3D) (*31*).

A total of 376 genes were classified as more likely to be repressed by FOXL2 because they were up-regulated in its absence. This group included genes encoding for signaling molecules crucial for ovarian development whose expression was shown to be independent of the FOXL2 pathway; therefore, these can be attributed to mechanisms that compensate for FOXL2 when it is deleted (*18*). These included *Rspo1* (fig. S1E) (*32*), and *Wnt4* (fig. S3E) (*33*) and exhibited stage-specific binding of FOXL2. In addition, we identified genes important for testis development such as *Gadd45g* (fig. S3F) (*34*) and *Inhbb* (fig. S3G) (*35*).

Last, we evaluated FOXL2 binding in the genomic region containing *Sox9*, a gene critical for Sertoli cell and testis development (fig. S3H) (*5*, *36*). We did not detect binding of FOXL2 to the *Sox9* promoter above background at any time point. We then scanned the 1-Mb gene desert upstream of *Sox9*, which was previously found to contain cis-regulatory elements essential for *Sox9* expression in Sertoli cells (*37*, *38*). We detected one significant peak at E14.5, two at 1W, and eight at 8W (fig. S3I). As previously shown (*6*, *39*), neither Testis-specific Enhancer of *Sox9* Core element (*TESCO*) enhancer nor Enhancer 13 (*Enh13*) was bound by FOXL2. Instead, we observed dynamic binding of FOXL2 to other known *Sox9* enhancers (*37*). For example, FOXL2 was bound to *Enh33* at 1W and to *Enh32* at E14.5. Significant binding events of FOXL2 to the ovary-specific *Sox9* *Enh8* (*37*), *Sox9* *Enh2*, and *Sox9* *Enh6* were detected at 8W. The remaining five FOXL2 binding events, four of which were detected at 8W and one at 1W, were distributed in other regions, not previously identified as Sertoli-specific *Sox9* enhancers. These may constitute granulosa-specific enhancers of *Sox9* used by FOXL2 to repress this gene in the ovary. We then clustered the ChIP-seq peaks into sets that showed similar patterns of variation in FOXL2 occupancy across the time course (Fig. 1D) and performed Gene Ontology (GO) analysis on each cluster (Fig. 1E and data file S1) to investigate which biological processes (BPs) FOXL2 regulates across ovarian development.

FOXL2 target genes known to be important in gonadal development were identified in each cluster. Those classified as shared included granulosa-enriched genes *Cited2*, *Smad7*, *Fst*, *Thbs1*, and the Sertoli-specific genes *Inhbb* and *Fgf9*. *Rspo1*, which encodes for a secreted signaling molecule that regulates wingless-type MMTV integration site family member 4 (WNT4) expression and is essential for female sex determination (*40*), was uniquely regulated by FOXL2 at E14.5, together with other genes including *Adora1*, *Pdgfc*, *Bcl9*, and *Tert*. These were enriched in cell migration and proliferation, cell projection organization, and Wnt signaling. Among the targets significantly bound by FOXL2 at 1W, we found genes involved in urogenital system development including *Amh*, *Fgfr2*, and *Ctnnb1*.

Among the genes uniquely bound by FOXL2 at 8W, we found an enrichment in those implicated in steroidogenesis pathways, including *Cyp19a1* encoding for aromatase (*41*), a known FOXL2 target (*39*); *Nr5a1*, encoding for SF1 and found previously as a FOXL2 partner (*27*); and *Hsd17b7*, involved in estradiol synthesis. While the cluster E14.5-1W shared several targets involved in urogenital development, the 1W-8W group featured genes encoding for members of the steroidogenesis pathway, including the known FOXL2 targets *Esr2*, for which we detected binding in the functional enhancer “*Peak 3*” (fig. S1D, marked with an asterisk) (*39*); *Star*, which encodes steroidogenic acute regulatory protein (*42*); and *Cyp17a1*. In addition, the 1W-8W cluster included sex differentiation genes such as *Wt1* (*43*), *Esr1* (*44*), *Irx3* (*45*), and *Wnt4* (*33*), as well as Sertoli-specific genes, such as *Sox9* (*36*), *Inha* (*46*), and *Fndc3a* (*47*). Last, the E14.5-8W cluster was enriched for DNA repair pathways, a recently discovered function of FOXL2 (*48*) and included genes such as *Ddx1*, *Ruvbl1*, and *Exo5*. We also identified an enrichment within this cluster of pathways not previously linked with FOXL2, including ncRNA processing and RNP complex biogenesis.

Next, we assessed the relative contribution of FOXL2 to the regulation of granulosa/female gonad–enriched genes versus Sertoli/male gonad–enriched genes. To obtain genes enriched in granulosa or Sertoli cells relevant for the embryonic and the postnatal stages of our time course, we used datasets from two studies (fig. S4A and data file S2): one by Zhao *et al.* (*49*) reporting comparison by RNA-seq of E13.5 XX (*X^GFP^X*) and XY embryonic gonads and another by Lindeman *et al.* (*50*) reporting RNA-seq analysis of primary granulosa cells from mice aged 23 to 29 days compared to Sertoli cells isolated from *CAG-Stop^flox-tdTomato^;DhhCre* P7 XY pups. In this model, *Dhh-Cre* activity is specific to Sertoli cells from E12.5, allowing this cell type to be marked early on (*51*). We then grouped all genes found to be enriched in female gonads by Zhao *et al.* (*49*) and added them to those identified as granulosa-specific postnatally by Lindeman *et al.* (*50*). We repeated the process for the male gonads/Sertoli-enriched genes and obtained two groups of markers of either ovaries or testes at embryonic and postnatal time points to which we compared the FOXL2 targets identified by our ChIP-SICAP across the time course.

Using this approach, we found that, at E14.5, 28% of FOXL2 targets were classified as either granulosa-specific or female enriched. This percentage increased for 1W targets (37%) and for 8W targets (36%). These comparisons, together with the overall greater number of targets identified at postnatal time points in our ChIP-SICAP, support a more prominent role of FOXL2 in regulating female/granulosa-specific genes postnatally.

### Identification of FOXL2 on-chromatin protein interactors

We next used ChIP-SICAP to identify on-chromatin protein interactors (chromatome) of FOXL2 by applying mass spectrometry on chromatin samples isolated at different stages of ovarian development (Fig. 1A). Proteins colocalizing with FOXL2 on the same chromatin fragments were pulled down using a specific antibody against FOXL2 followed by DNA biotinylation to separate chromatin-bound proteins.

**Fig. 2.**
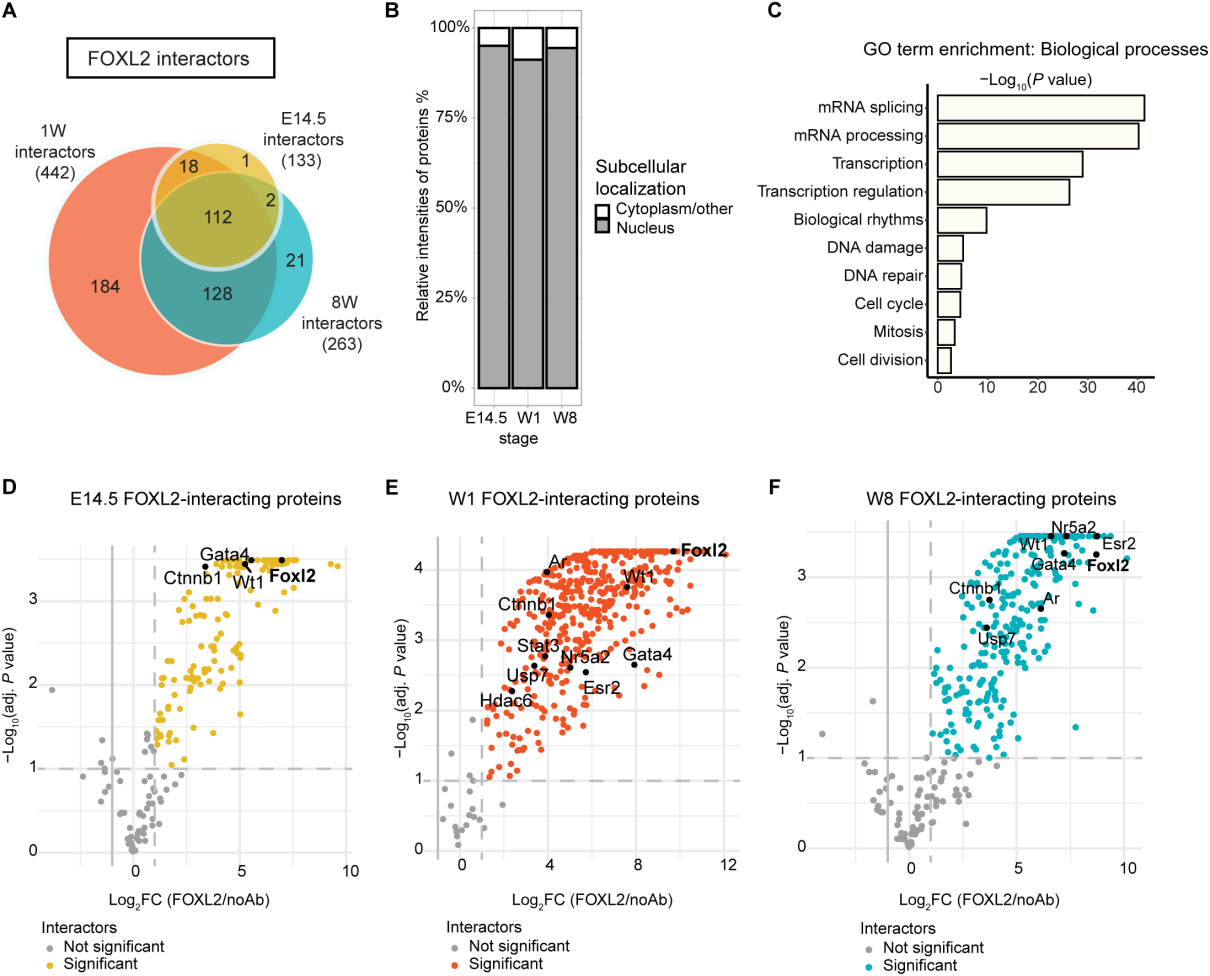
Identification of FOXL2 on-chromatin interactors by ChIP-SICAP. (**A**) Venn diagram representing the FOXL2 interactors identified by FOXL2 ChIP-SICAP. The total number of proteins is reported between round brackets. (**B**) Bar chart showing the relative intensities calculated using iBAQ (intensity-based absolute quantification) of the proteins identified and classified as either nuclear or cytoplasmatic only (proteins shuttling between nucleus and cytoplasm are classified as nuclear). (**C**) GO BP enrichment analysis. (**D** to **F**) Volcano plot showing enrichment [Benjamini-Hochberg (BH) adjusted *P* < 0.1 and mean fold enrichment > 2] of FOXL2 immunoprecipitate versus no antibody control (*n* = 2 biological replicates/time point) of proteins colocalizing with FOXL2 on chromatin at E14.5 (D), 1W (E), and at 8W (F). Dotted lines separate significant proteins, also highlighted in yellow, orange, and blue. Black dots, selected FOXL2 interactors.

The two-step purification strategy improves the purity of the samples by reducing the artificial interactions that are generated during the immunoprecipitation procedure and removes non-chromatin bound proteins. We identified a total of 133 proteins at E14.5, 442 at 1W, and 263 at 8W (>2–fold change FOXL2/no antibody control, *n* = 2, adjusted *P* < 0.1; Fig. 2A and data file S3).

Comparison of the relative protein intensities showed that the most highly abundant proteins were known nuclear proteins, indicating a successful enrichment for nuclear factors over potential cytoplasmic contaminants (Fig. 2B). GO enrichment analysis of FOXL2 interactors showed enrichment of proteins involved in splicing, transcription, DNA replication and repair, and cell cycle (Fig. 2C and fig. S4). FOXL2 was among the top 20 mostly enriched proteins identified [based on log2 fold change (log_2_FC), Fig. 2, D to F, and data file S3]. We also identified previously known FOXL2 nuclear interactors, namely, signal transducer and activator of transcription 3 (STAT3), suppressor of Ty 16 homolog (SUPT16H), alpha-thalassemia/mental retardation, X-linked (ATRX), SWI/SNF-related matrix-associated actin-dependent regulator of chromatin subfamily D member 2 (SMARCD2), and x-ray repair cross complementing 6 (XRCC6) (*52*). Among the FOXL2 protein partners present across the time course, we found CTNN1B (β-catenin), a member of the WNT signaling pathway controlling the differentiation of the mouse ovary, Wilms’ tumor suppressor (WT1), a TF essential for the development of the bipotential genital ridge and for the formation of the adult reproductive systems in both sexes (*53*, *54*), but where high levels of the −KTS (lysine, threonine, and serine) isoforms drive ovary differentiation (*55*), factors important for early gonadal development and folliculogenesis such as GATA4 (*56*, *57*), cell cycle regulators such as DNMT3A, heterochromatin-interacting proteins such as CBX3, and members of the splicing system including the DNA helicase DHX9, SRSF1, and DDX17. Throughout the time course, we also detected the E3-SUMO ligase TRIM28 that we previously showed to cooperate with FOXL2 in maintaining ovarian cell fate (*6*).

We identified a series of FOXL2-interacting proteins, with well-recognized roles in ovarian development, PFA, and folliculogenesis in humans and mice, being differently present across the time course (Fig. 2, D to F). We detected nuclear receptor subfamily 5 group A member 2 (NR5A2) (*58*) colocalizing with FOXL2 at 1W and 8W. In addition, we found two known regulators of the primordial-to-primary follicle transition at 1W, namely, the histone deacetylase 6 (HDAC6) (*59*) and the TF STAT3 (*60*).

Androgen receptor (AR), a factor that contributes greatly to normal follicle development (*24*), was enriched at 1W and 8W but not earlier at E14.5. We identified estrogen receptor 2 (ESR2) among the FOXL2 interactors at both postnatal time points, in agreement with the previously postulated interplay between these two factors in orchestrating key functions in ovarian physiology such as steroidogenesis (*39*) and cell fate maintenance (*7*).

We then compared our in vivo FOXL2 chromatome with a FOXL2 co-immunoprecipitation, proteomics dataset performed on the whole-cell granulosa-like cell line AT29C (*52*) to assess the level of overlap between our chromatin-specific dataset and one that would also include cytoplasmic interactors. We found that 112 of the 464 proteins that we identified throughout the time course were also found in this study. Of these shared FOXL2 interacting partners, 36 were found in our E14.5 time point, 109 at 1W, and 63 at 8W (fig. S5B and data file S3). These shared proteins included the known FOXL2 interactor involved in sex maintenance TRIM28 (*6*); chromatin remodeling enzymes such as ATRX, which is involved in sexual differentiation (*61*); regulators of the WNT signaling pathway such as CXXC5 and USP7; and DNA repair proteins such as XRCC6, previously identified in a yeast two-hybrid screen and co-immunoprecipitation study (*62*). Within this shared set, we also detected an enrichment of splicing factors colocalizing with FOXL2. These included, for example, SRRT, SF3A1, RBMX, and WTAP1.

In summary, we showed that FOXL2 differentially interacts with proteins playing a role in a range of gonadal-essential functions during ovarian differentiation, from gonadogenesis, through PFA, to steroidogenesis, as well as other interactors with more general roles in chromatin remodeling, DNA repair, and splicing.

### Generation of a *Foxl2^EGFP^* mouse line to isolate granulosa cells

**Fig. 3.**
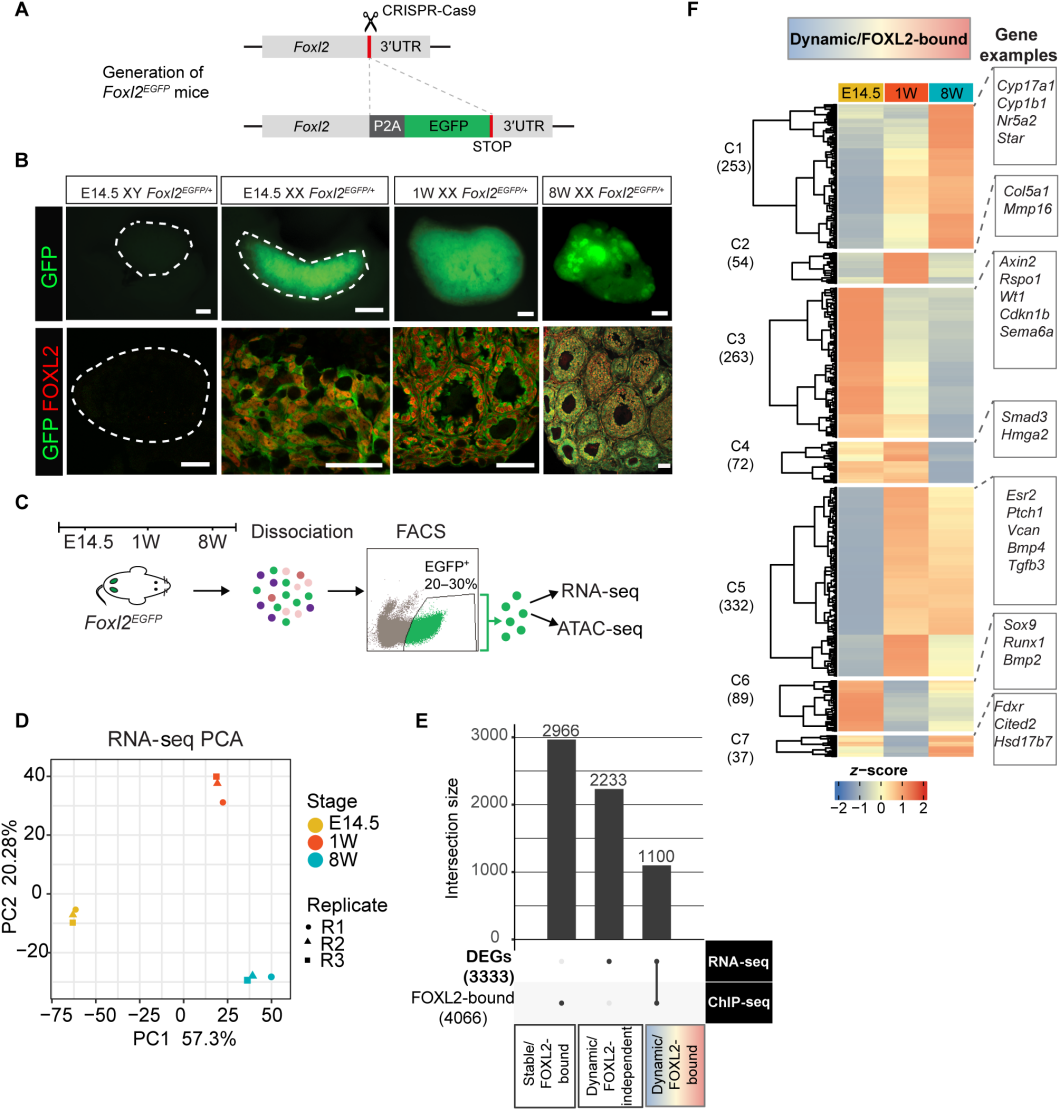
An integrative approach combining RNA-seq on *Foxl2^EGFP^* granulosa cells and ChIP-seq refines the GRNs controlled by FOXL2. (**A**) CRISPR-Cas9 mediated enhanced green fluorescent protein (EGFP) insertion at the *Foxl2* locus. 3′UTR, 3′ untranslated region. (**B**) Top row: Representative images of EGFP fluorescence in freshly collected gonads. Scale bars, 200 μm. Dotted lines outline the embryonic testis (left) and ovary (right). Lower row: colocalization of GFP and endogenous FOXL2 assessed by immunofluorescence, Scale bars, 100 μm. (**C**) Overview of sample collection and downstream analyses. (**D**) PCA plot of RNA-seq analysis of cells isolated from E14.5, 1W, and 8W gonads (*n* = 3 biological replicates). (**E**) Number of differentially expressed genes (DEGs) identified by RNA-seq, FOXL2 gene targets identified by ChIP-SICAP, and the overlap between the two datasets. DEGs identified by DESeq2’s Wald test, significant genes have an FDR < 0.01, a shrunken log_2_FC > 2 and a baseMean (i.e., mean abundance across all samples) > 2. (**F**) Hierarchical clustering of the 1100 genes bound by FOXL2 and differentially expressed across the time course. Gene examples are shown on the right. FDR < 0.05 (DESeq2 analysis of FOXL2 occupancy).

We next sought to analyze the transcriptome and chromatin landscape of granulosa cells expressing FOXL2, to ultimately integrate these datasets with the ChIP-seq data, thus reconstructing the gene regulatory networks (GRNs) at play throughout ovarian development. We used CRISPR-Cas9 genome editing to generate a mouse line expressing an enhanced green fluorescent protein (EGFP) reporter knocked into the *Foxl2* locus, which allowed the isolation of cells expressing functional FOXL2 (Fig. 3A and fig. S6, A to F). The line was maintained as heterozygotes. Fertility performance of *Foxl2^EGFP/+^* females was monitored over 12 weeks and found to be normal compared to control females (fig. S6G). Ovaries collected from heterozygous *Foxl2^EGFP^* (*Foxl2*^*EGFP*/+^) mice were imaged by fluorescence microscopy and showed a bright EGFP signal specifically in the ovaries at all stages examined (Fig. 3B and fig. S7A). As expected, given the known absence of FOXL2 expression in the testis, no EGFP signal was detected in the gonads of XY *Foxl2*^*+*/+^ or *Foxl2^EGFP/+^* males and *Foxl2^+/+^* XX control ovaries. In addition, in agreement with previous studies that investigated the expression of a *Foxl2^LacZ^* reporter transgene (*63*, *64*), we observed EGFP expression in the uterus, developing eyelids, in cranial mesodermal cells and cranial neural crest cells of the developing embryo, and in the pituitary gland (fig. S7, B to D) (*65*).

Colocalization of EGFP with the endogenous FOXL2 protein in *Foxl2^EGFP/+^* ovaries was confirmed by immunofluorescence throughout ovarian development (Fig. 3B and fig. S8).

We dissociated ovaries from *Foxl2^EGFP/+^* mice at E14.5, 1W, and 8W (Fig. 3C and fig. S7B) and isolated green fluorescent protein (GFP)–positive cells by fluorescence-activated cell sorting (FACS). A total of 50,000 and 100,000 *Foxl2^EGFP^*-positive cells were processed for bulk RNA-seq and ATAC-seq, respectively, using three biological replicates for each time point.

PCA of the RNA-seq data (Fig. 3D) based on the variance-stabilized (VST) abundance estimates showed good separation between the time points indicating major transcriptional changes characterizing granulosa *Foxl2^EGFP^-*positive cells throughout time. Most of the variance was evident along the first principal component, which separated E14.5 samples from both W1 and W8. Poisson similarity metric indicated more similarity between the transcriptome of postnatal samples (1W and 8W) compared to E14.5 (fig. S10A). To examine the global transcriptome dynamics, we selected genes significantly changing across the time course in at least one pairwise comparison (data file S4). Differential gene expression analysis comparing 8W and E14.5 revealed the greatest changes with 1436 genes up-regulated and 1134 down-regulated; comparing 1W with E14.5 revealed up-regulation of 1307 genes and down-regulation of 861, while the least number of changes was detected between 8W and 1W, with 489 genes up-regulated and 556 genes down-regulated (fig. S10B). Because FOXL2 is also expressed, albeit weakly, in theca cells (*66*, *67*), we first assessed the expression values of known theca and granulosa cell markers (*68*). We found an enrichment of granulosa over theca markers, confirming the suitability of this dataset for the exploration of granulosa-specific GRNs (fig. S10C). We found that FOXL2 did not strongly colocalize with HSD3β^+^ theca cells. It should be noted that theca cell nuclei were not expressing endogenous FOXL2 as strongly as granulosa cells. In addition, the CYP17A1-positive androgen-producing theca cells (*68*) were negative for GFP. We then used *k*-means clustering to analyze the dynamics of gene expression changes across time (fig. S10, E and F) and identified seven main patterns. Clusters 7 and 6 consisted of genes highly expressed at E14.5, including markers of pre-granulosa cells such as *Rspo1*, *Lgr5*, and *Wnt4*, important for the initial cell proliferation in XX embryonic gonads (*30*), which were down-regulated at postnatal time points. GO term BP analysis (fig. S11) of these clusters revealed enrichment of BPs such as Wnt signaling and urogenital system development pathways. Cluster 5 (C5) included genes moderately expressed at E14.5 and up-regulated specifically at 1W and included genes involved in kidney and urogenital system development, genetic imprinting, and extracellular matrix organization. C1 consisted of genes uniquely up regulated at 1W and participating in extracellular matrix organization, cell cycle phase transition, and DNA replication. C4 grouped genes activated as the ovary develops from 1W and remain up-regulated in adulthood at 8W. GO analysis showed enrichment in nuclear division, cell cycle, lipid transport, and synapse organization. Last, C3 included genes specifically up-regulated at 8W and enriched in steroid metabolic processes and angiogenesis. These data show the timely activation of genes important for embryonic and postnatal ovarian development and function in our system.

### An integrative approach to reconstruct GRNs controlled by FOXL2

Having identified the gene expression changes characterizing *Foxl2^EGFP/+^* granulosa cells, we then sought to reconstruct the GRN underpinned by FOXL2 (Fig. 3E and data file S5).

We took the total number of FOXL2-bound genes identified by ChIP-seq at any given time point (Fig. 1) and intersected it with the set of differentially expressed genes (DEGs) identified by RNA-seq. Genes that changed in their expression value at any point between E14.5, 1W and 8W (3333 genes) and that were assigned to a FOXL2 ChIP peak in at least one time point (4066 genes) were considered as “dynamic/FOXL2-bound” genes. The rest of the genes bound by FOXL2 but not changing their expression values were considered as “stable” (2966 in total) and divided between “stably repressed” and “stably activated,” based on a VST cutoff of 8.3 (fig. S12, A and B).

This threshold was chosen on the basis of the expression values of known granulosa-enriched genes (*Foxl2*, *Esr2*, and *Rspo1*) versus genes residing on the Y chromosome therefore not expected to be expressed at all in XX ovaries (*Sry*, *Usp9y*, and *Uty*), as well as on the expression values of Sertoli-enriched genes (*Dmrt1*, *Sox9*, and *Fgf9*) (fig. S12C). Using this approach, we identified a total of 777 genes bound by FOXL2, but which were not expressed at any point during the time course and are therefore assumed to be repressed by FOXL2 (fig. S13A). These were enriched in cell-cell adhesion, immune, and protein secretion pathways (fig. S13B). These also included Sertoli-enriched genes such as *Spz1* (*69*), *Cdh8*, *Rims1*, *Fgf9* (*70*), *Hsd17b3*, *Hopx*, and *Tuba3* (*50*). A higher number of genes were bound by FOXL2 and were expressed throughout the time course but did not significantly change in levels across this (2189 genes). These were enriched in mRNA processing, ubiquitin-dependent catabolic processes, cell cycle, and histone modification pathways. Last, the dynamic/FOXL2-bound group contained a total of 1100 genes. Unsupervised hierarchical clustering identified seven main clusters (Fig. 3F). GO enrichment analysis (fig. S13C) of these clusters showed that FOXL2 regulates a plethora of dynamically expressed genes enriched for functions critical for different stages of ovarian development. For example, we found enrichment in steroid metabolic and angiogenic processes, typical of folliculogenesis, in C1, which included genes up-regulated specifically at 8W. Of these, 53% were also a direct target of FOXL2 specifically at 8W (data file S5). On the contrary, if considering C3, C4, and C6, clusters included genes up-regulated at E14.5 compared to 8W, only 12% of the genes were significant targets of FOXL2 at E14.5. This confirmed the preponderant role of FOXL2 in regulating significantly changing genes at postnatal stages rather than during embryonic development. The high enrichment of Wnt signaling pathway genes found in C3, which contained a total of 263 genes up-regulated at E14.5 only 36 of which were bound by FOXL2 at this time point (including several Wnt signaling pathway effectors such as *Rspo1*, *Zfp703*, and *Bcl9*), was consistent with the established role of this pathway in ovarian development (*33*). Of note, C6 contained *Sox9*, for which we detected low levels of gene expression at E14.5 before being down-regulated at postnatal time points.

### *Foxl2^EGFP^*-positive cells have a dynamic chromatin landscape across ovarian development

**Fig. 4.**
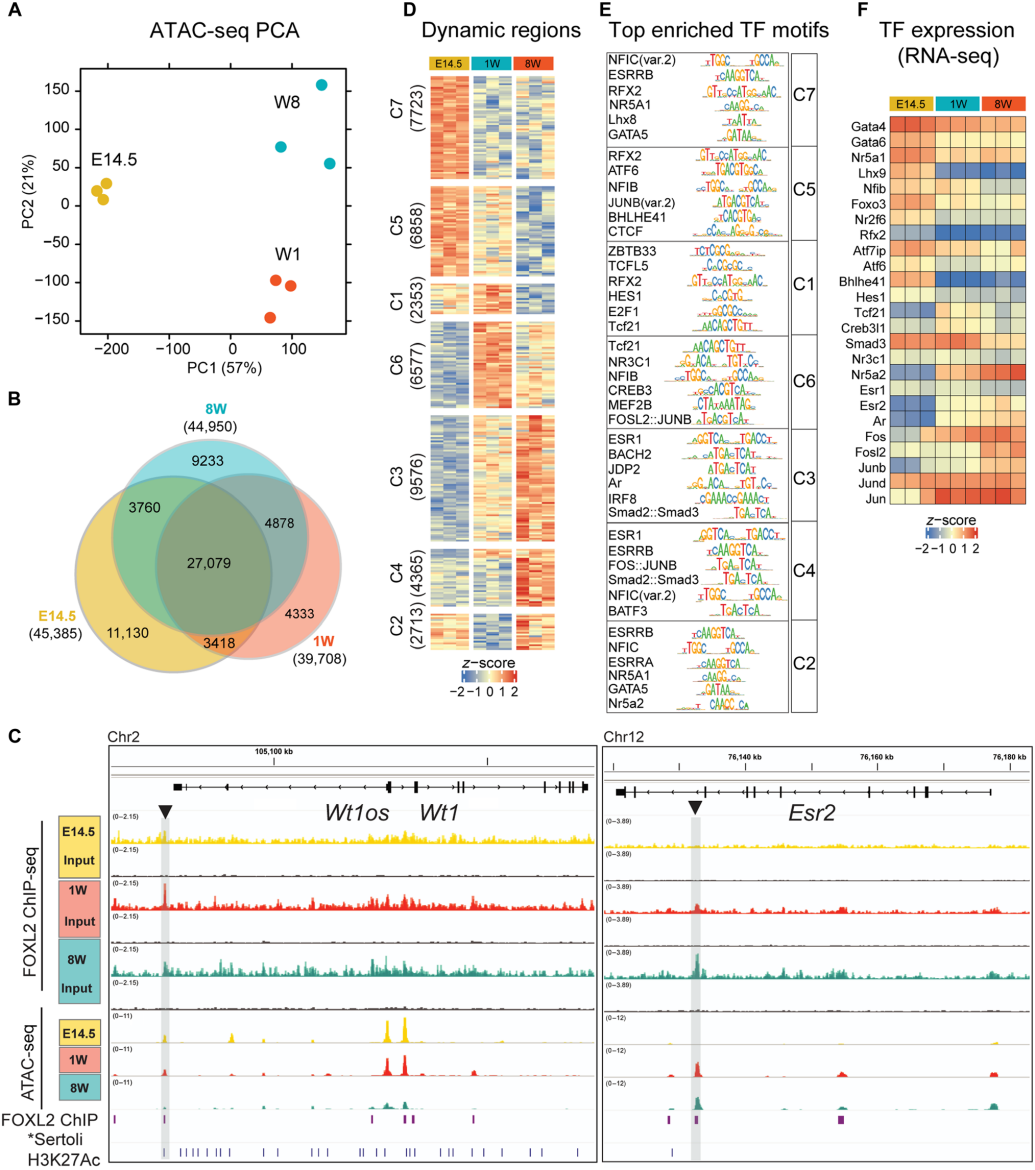
Granulosa cells have a dynamic chromatin landscape throughout development. (**A**) PCA of ATAC-seq performed on *Foxl2 ^EGFP/+^* sorted somatic cells at E14.5, 1W, and 8W (*n* = 3 biological replicates, consensus peaks identified by DiffBind, FDR < 0.05, and assigned to the nearest gene by ChIPpeakAnno). (**B**) Venn diagram reporting the number of significant peaks. (**C**) Hierarchical clustering of peaks using Euclidean distance metric and complete linkage. (**D**) monaLisa analysis of TF motifs enrichment within the open chromatin regions. Top six motifs for each cluster are reported (data from table S6). (**E**) Heatmap displaying gene expression changes of representative TF, as assessed by RNA-seq (data from table S4). (**F**) Integrative Genomics Viewer (IGV) snapshots of representative regions and corresponding ATAC-seq and ChIP-SICAP peaks. *Sertoli H3K27Ac ChIP-seq was reanalyzed to match mm10 coordinates from (*67*)*.* Gray bars and black arrowheads highlight significant peaks over input as assessed by ChIP-SICAP.

To explore the dynamics of chromatin remodeling in granulosa cell development, we performed ATAC-seq on *Foxl2^EGFP^-*positive cells (Fig. 4A and data file S6).

In total, we identified 45,385 accessible regions at E14.5, 39,708 at 1W, and 44,950 at 8W (Fig. 4B and fig. S14A). Of these, 27,079 were accessible throughout the time course, while 11,130 were unique to E14.5, 4333 unique to 1W, and 9233 unique to 8W. The genomic annotation of open chromatin regions was similar across time points, with 32 to 35% mapping to promoters, 34 to 38% to the gene body, 3% downstream of a gene, and 25 to 28% mapping to distal intergenic regions (fig. S14B). *K*-means clustering of consensus peaks revealed seven patterns representing similar dynamic opening and closing of chromatin regions (fig. S14C). To assess the potential contribution of FOXL2 to binding to these regions, we specifically looked for enrichment values for the FOXL2 canonical motif (JASPAR id: MA1607.1) in each ATAC-seq cluster (fig. S14D). This analysis revealed a greater enrichment in C6, C3, C4, and C2 and the least in C7 (fold enrichment of 1.4 to 1.2, adjusted *P* > 4). Among these, C6 and C3, which included peaks accessible preferentially at 1W and 8W and closed at E14.5, were the two most significantly enriched with FOXL2 motifs. In contrast, among regions accessible at E14.5 (C1, C5, and C7) only C7 contained significant enrichment of FOXL2 binding sites, albeit with an enrichment score much lower than C6 and C3.

We performed differential accessibility analysis using DESeq2 (data file S6) (*71*) and identified a total of 40,165 differentially accessible regions (DARs) throughout the time course, 32,611 of which were changing between E14.5 and 8W, 24,026 were changing between E14.5 and 1W, and 16,054 were changing between 1W and 8W. These chromatin landscape changes were consistent with the transcriptomic data, which showed that most of the differences were detected comparing E14.5 and 8W samples. DARs included potential cis-regulatory regions, for example, one within the *Rspo1* gene, conserved, and differentially bound by FOXL2 according to our ChIP-SICAP analysis, and showing a similar change in its accessibility, as indicated by ATAC-seq (fig. S14E). We also detected FOXL2 binding by ChIP-seq at a known *Wt1* enhancer located 50 kb upstream of its promoter (Fig. 4C) (*72*). This binding correlated with closing of this region at 8W. In contrast, *Esr2* was bound by FOXL2 at its known functional enhancer (*39*) only at postnatal stages. This enhancer was not marked by H3K27Ac (active enhancer mark) in Sertoli cells and, therefore, can be considered as a granulosa-specific cis-regulatory region of *Esr2*.

*K*-means clustering identified seven clusters representative of the main chromatin remodeling patterns (Fig. 4D). Among the DARs, we detected regions located nearby granulosa cell markers such as *Fst* (*73*) and *Runx1* (*8*) that were highly conserved (fig. S14E). In addition, we identified open elements nearby markers of Sertoli cells, including structural markers such as *Cldn11* and *Itga6*, hormonal markers such as *Amh* and *Hsd17b1*, and genes encoding for TFs such as *Nr5a1* and *Fgf9*. These were also bound by FOXL2 as assessed by ChIP-SICAP.

To investigate what TFs may bind to these dynamic regions that could potentially drive the observed gene expression changes or that may direct changes in chromatin organization, we performed a TF motif enrichment analysis of the DAR dynamics using monaLisa (Fig. 4E and data file S5) (*74*). Regions that were more accessible at E14.5 but lost accessibility at later stages (namely, C7 and C5) were enriched with nuclear factor family motifs and GATA motifs. NR5A1 motifs were highly represented, as well as motifs from the RFX family. C5 was also enriched in motifs typical of the transcriptional repressor CTCF. Regions accessible at 1W (C1 and C6) were enriched in motifs for factors involved in Wnt signaling (TCFL5 and TCF21) and Notch signaling (HES1) and for ZBTB33 motifs, a transcriptional repressor also known as Kaiso, which negatively regulates the Wnt signaling pathway (*75*). Among these, TCF21 and ATF1 were also found to localize with FOXL2 on chromatin postnatally, as found by FOXL2 ChIP-SICAP (data files S3 and S5). Clusters containing regions opened at 8W were enriched with motifs for members of the steroid hormone receptor family, including estrogen related receptor beta (ESRRB, containing a half-site for the binding of ESR2) and AR, factors known to play critical roles in folliculogenesis and ovulation (*24*, *76*), which were also found in our proteomics analysis (Fig. 2). ESRRB motifs were also enriched at E14.5 although no expression of *Esr2* mRNA was found at this time point by RNA-seq (Fig. 4F). Last, in C2, we found enrichment for the TF NR5A2, known to regulate PFA (*58*), which was also detected with FOXL2 ChIP-SICAP at 1W and 8W time points.

RNA-seq analysis revealed that some of these factors also show an expression pattern matching the binding motif predictions. For example, *Gata4* and *Lhx9* expression was increased at E14.5, while factors predicted to bind at postnatal stages including *Nr5a2*, *Ar* and *Esr2* were up-regulated from 1W onward.

### Deletion of *Usp7* in somatic cells blocks granulosa cell differentiation and impairs ovarian development

To select for previously unknown players controlling folliculogenesis, we focused on proteins that were bound with FOXL2 on chromatin at 1W and 8W, when PFA occurs and follicles initiate the transition toward growing follicles, but not at E14, and that were associated with any of the following terms in OMIM database: “premature ovarian failure,” “hypogonadisms,” or “infertility,” to enrich for factors more likely to be relevant to human conditions. We found one protein named USP7 (Fig. 2, E and F) satisfying these criteria, where individuals carrying gene mutations exhibited developmental delay, intellectual disability, and hypogonadism in both sexes (*77*, *78*). USP7 is recognized to play a role in chromatin regulation directly through the depletion of ubiquitin marks involved in DNA methylation (*79*) and indirectly by controlling the stability of DNA methyltransferases (*80*, *81*). USP7 is also a regulator of Wnt signaling, a pathway with a pivotal role in pre-granulosa cell activation during PFA (*82*). We checked the expression of *Usp7* in our RNA-seq experiment on FOXL2-GFP–positive cells and found that it had an expression profile very similar to that of *Foxl2* itself (fig. S15A).

To test the function of USP7 in granulosa cells, we conditionally deleted *Usp7* (*83*) using *Sf1:Cre^tg/+^* transgenic mice (*84*). *Usp7^fl/+^;Sf1:Cre^tg/+^* mice of both sexes did not display any obvious morphological or reproductive phenotypes. We then analyzed the phenotype of *Usp7^fl/fl^* mice lacking the Cre driver transgene, defined herein as control, and compared it to those of *Usp7^fl/fl^;Sf1:Cre^tg/+^* mutants.

**Fig. 5.**
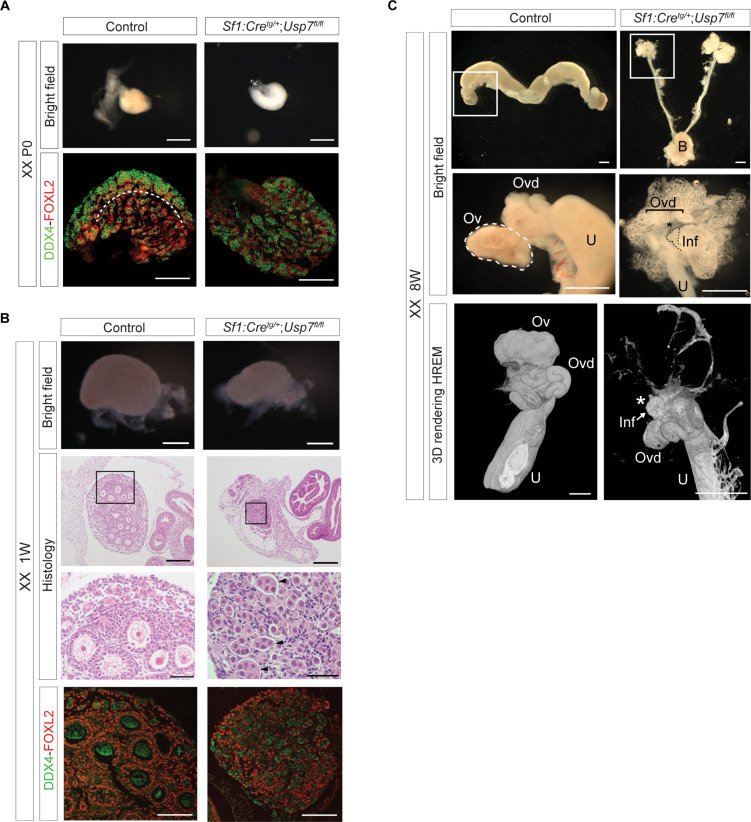
Deletion of *Usp7* blocks somatic cell differentiation and impairs ovarian development. Characterization of ovaries collected from *Usp7^fl/fl^* mice (control) and *Usp7^fl/fl^;Sf1:Cre^tg/+^* mice (mutants). Representative images of at least *n* = 3 biological replicates. Scale bars, 500 μm in all, unless otherwise specified. (**A**) Top row: Bright-field images showing morphology of control and mutant P0 ovaries. Bottom row: Immunofluorescence analysis of germ cell marker DDX4 (green) and granulosa cell marker FOXL2 (red) in ovary sections. Scale bars, 50 μm. (**B**) From the top: Bright-field images of 1W ovaries; hematoxylin and eosin staining; scale bars, 200 μm; higher magnification showing persistence of nest-like structures in mutants (arrowheads; scale bars, 50 μM) and absence of primary and secondary follicles; immunofluorescence staining for DDX4 and FOXL2 in 1W ovaries. (**C**) Reproductive tracts and ovaries from 8W control and mutant mice. Higher magnification; scale bars, 1 mm. Last row: High-resolution episcopic microscopy (HREM) three-dimensional (3D) rendering of reproductive tract from an 8W control ovary and a XX mutant. Asterisk indicates the area where an ovary should be. Arrow indicates infundibulum tip and lack of ovarian structure. Scale bars, 0.7 mm. Ovd, oviduct; Ov, ovary; U, uterus; B, bladder; Inf, infundibulum.

In homozygous mutants, no evident morphological abnormalities were observed when comparing ovaries to control mice at P0 (Fig. 5A). However, immunofluorescence analysis showed that the clear boundary between the cortex and medullary region of the ovary, present in the controls, was lost in mutant ovaries. FOXL2/DDX4 double staining revealed that, in control ovaries, germ cells were densely packed in the cortical region, while individual oocytes surrounded by FOXL2-positive granulosa cells started to organize themselves into what would later develop as individual follicles in the medulla. In contrast, mutant ovaries did not display such demarcation, granulosa cells appeared disorganized in their spatial localization, and most oocytes remained in germ cell nests. Immunofluorescence double staining for FOXL2 and USP7 in ovaries collected at P0 from control mice showed that FOXL2 colocalize with USP7-expressing somatic cells of the gonad (fig. S15A). Of note, USP7 was highly expressed in oocytes.

At 1E postnatally, we observed that mutant ovaries were visibly smaller compared to controls (Fig. 5B). Control ovaries contained a cortex of primordial follicles, where dormant oocytes were surrounded by one layer of flattened granulosa cells, and a medullary region, containing follicles which had been activated and progressed toward the primary follicle stage. In these, granulosa cells were well organized, with some having completed the transition from flattened to cuboidal and strongly expressing FOXL2. Notably, in mutant ovaries, only primordial follicles were found. In addition, several nest-like structures were identified encapsulating clusters of germ cells, which are not expected at this age. Granulosa cells in *Usp7^fl/fl^;Sf1:Cre^tg/+^* mice were flattened, expressed FOXL2, and appeared to be arrested at the primordial follicular stage. At this stage, USP7 colocalized with FOXL2 in controls, similarly to the P0 time point; while in mutants, it was not detected in the somatic cells (fig. S15B). These experiments confirm successful ablation of USP7 specifically within the somatic cell component of the gonads. Overall, these data indicate that expression of USP7 in the somatic cell lineage is necessary for granulosa cell differentiation and proliferation as no follicle beyond the stage of primordial was identified in mutants.

In adults, no ovarian structures could be found in 8-week-old *Usp7^fl/fl^;Sf1:Cre^tg/+^* mice, and the oviduct terminated with the infundibulum tip (Fig. 5C and movie S1), indicating complete degeneration of the ovary in the absence of *Usp7*. These results indicate that USP7 must also be essential for the survival of the somatic cell populations of the ovary. The uterus of 8-week-old mutant mice was markedly hypoplastic, consistent with hypoestrogenism expected to occur consequently to the loss of ovarian function. Because *Usp7* was reportedly found mutated in XY patients exhibiting hypogonadism (*77*, *78*), we also investigated the phenotype of mutant testes. *Usp7^fl/fl^;Sf1:Cre^tg/+^* testes were smaller than controls from P0 (fig. S16A). Immunofluorescence staining revealed an increased number of DDX4-positive germ cells in *Usp7^fl/fl^;Sf1:Cre^tg/+^* mice compared to control littermates. The size difference between control and mutant gonads was accentuated at 1W postnatally (fig. S16B), when testes started to become markedly hypoplastic. Immunofluorescence for SOX9 revealed abnormal distribution of Sertoli cells and disorganized tubules. The mutants often presented with a fluid-filled cavity lined by a SOX9-negative epithelium. The hypoplasia of mutant testes was more pronounced in adult gonads, when the testes of mutant mice exhibited a 98% decrease in their weight (fig. S16C, left). Homozygous mutants were completely sterile (fig. S16C, right). Mature elongated spermatids were absent in mutants (fig. S16D), while these were found in controls. Tubules were dysgenic, showing a reduction in the number of Sertoli cells and often exhibiting depletion of germ cells from defective tubules.

These results suggest that USP7 is necessary for the correct specification and function of somatic cell lineages in both the ovary and the testis and for fertility in both sexes. FOXL2 is likely to be a critical interacting factor for USP7 in the ovary, but other partners may be present in the testis where FOXL2 is not expressed and interact with USP7 in this context.

## DISCUSSION

In this study, we describe the set of FOXL2 genomic targets, on-chromatin protein interactors, gene expression changes, and chromatin landscape dynamics characterizing granulosa cells at three critical stages of mouse ovarian development. We found that FOXL2 binds to a greater number of genes at postnatal compared to embryonic stages, supporting a more critical role for this factor during postnatal life compared to the embryo, at least in mice. GO analysis of FOXL2 targets at E14.5 showed enrichment of Wnt signaling, in keeping with the synergistic role played by these two pathways in reinforcing ovarian cell fate commitment (*85*). The Wnt pathway has been shown as essential for pre-granulosa cell activation during PFA (*82*). This pathway was also highly featured among FOXL2 target genes identified at 1W, supporting the idea that a possible interplay between Wnt and FOXL2 around the time of PFA may happen through regulation of similar targets. Our time course analysis coupled with RNA-seq showed that two genes crucial for Wnt signaling, namely, *Rspo1* and *Wnt4*, were down-regulated as the ovary develops, with *Rspo1* being down-regulated below the threshold, while *Wnt4* expression was gradually turned down but not completely abrogated. From our ChIP-SICAP, it can be noted that the binding pattern of FOXL2 to these genes followed their expression pattern. That is, FOXL2 binds to *Rspo1* when this gene is expressed at E14.5 before disengaging at 1W and 8W when it is down-regulated; in addition, FOXL2 is bound to an intronic putative enhancer of *Wnt4* throughout the time course, showing a larger binding peak at postnatal time points. Our results therefore suggest that, in contrast to what was previously thought, FOXL2 may have a direct role in regulating the RSPO1/WNT4 pathway. These genes were also found bound by FOXL2 by another previously published study by Nicol *et al.* (*29*), which has investigated the genomic binding of FOXL2 by ChIP-seq at E14.5. Although our studies have applied different techniques and antibodies against FOXL2 proteins and, therefore, may not be directly comparable (*86*), when we examined the degree of overlap in specific regions of known FOXL2 targets, we found similar peak enrichment in genes with a role in granulosa cell identity, such as *Cited2* and *Fst*, or others likely to be repressed by FOXL2 in the ovary, such as *Fgf9*. Others, however, were missing in our dataset at E14.5, namely, *Dmrt1* and *Cited1* genes important in Sertoli cells. Our complete time course analysis provides further insights into the dynamic role of FOXL2 throughout ovarian development and offers evidence that it plays a greater role at postnatal stages.

Genes bound at 8W, once mice have reached sexual maturity, are enriched for steroidogenesis and DNA repair pathways. Notably, we found FOXL2 to directly bind to a known ESR2 enhancer (*39*), specifically at 8W. In addition to binding sites for forkhead factors, FOXL2 peaks were highly enriched in steroid receptor motifs including ESR1 and AR. This supports previous in vitro studies that indicated a role for FOXL2 on estradiol signaling (*39*).

One of the main roles of FOXL2 in the ovary is direct repression of *Sox9* (*7*). Our data show that this repression may be time-specific, as we detected a greater number of FOXL2 peaks in the “gene desert” regulatory region 5′ to *Sox9* locus at 8W, compared to earlier time points. However, this does not exclude that the presence of a single peak at earlier time points may suffice and act as repressor site for *Sox9*. We detected low levels of *Sox9* expression in GFP-positive cells at E14.5, which are then down-regulated at postnatal stages. This agrees with other transcriptomic studies which have also detected *Sox9* expression in pre-granulosa cells collected between E12.5 and E16.5 (*87*). The lower occupancy of FOXL2 within the *Sox9* gene desert at this time point, compared to an increased occupancy at postnatal stages, may explain the expression of this gene in early granulosa cells.

In agreement with other studies, we did not find binding of FOXL2 to *TESCO* (*6*, *11*, *29*) nor to the distal *Sox9* *Enh13* (*37*, *88*), but, instead, we detected peaks in other regions, mostly not overlapping with Sertoli-specific *Sox9* enhancers (*37*). In addition, FOXL2 appeared to bind to several Sertoli-enriched genes, with structural roles (*Cldn11* and *Itga6*) or involved in hormone signaling (*Hsd17b1*, *Amh*, and *Inhbb*). These data suggest that FOXL2 may repress testis development postnatally through alternative *Sox9* enhancers, many of which are granulosa-specific, but also by directly repressing genes important for Sertoli cell proliferation and function. The latter may well be the most important function of FOXL2 beyond repressing *Sox9*. This was suggested to be the case in the conditional TRIM28 null mutant XX gonads, where expression of Sertoli structural genes precedes *Sox9* up-regulation in an intermediate cell type along the female-to-male transdifferentiation trajectory (*6*).

Among the FOXL2 targets, we also identified enrichment in pathways not previously associated with FOXL2, including noncoding RNA processing and RNP complex biogenesis. Noncoding RNAs are starting to be recognized to have a role in sex determination, gonadal development, and folliculogenesis, and several studies have reported the sexually dimorphic expression of microRNAs during sex determination (*89*, *90*). In addition, it was found that genes necessary for testis determination and differentiation, including *Sry* and *Sox9*, as well as for ovarian development, including *Foxl2* and *Wnt4*, are among the predicted targets of differentially expressed miRNAs. Long noncoding RNAs that were implicated in folliculogenesis, are known to regulate the translation of proteins directly or indirectly, thereby inhibiting the proliferation of granulosa cells and affecting cell cycle progression (*90*, *91*), which has to be tightly regulated to ensure proper granulosa cell differentiation. In addition, RNP complex biogenesis was found to be enriched among the FOXL2 targets. An example of RNP complex is ribosomes, which are essential for protein synthesis, and their assembly is a fundamental rate-limiting step for cell growth and proliferation. Missense pathogenic variants involving the RNA helicase *Dhx37*, which is essential for ribosome biogenesis, have been found in 46,XY DSD (*92*). Another example of ribonucleoprotein complexes is spliceosomes, the cell machinery that regulates splicing events. This evidence together suggests additional regulatory roles may be indirectly played by FOXL2 through the regulation of noncoding RNA expression, protein translation, and splicing.

We then queried the nature of the binding partners that were colocalizing on the same chromatin regions with FOXL2. Our ChIP-SICAP analysis represents the first in vivo analysis of chromatin binding partners of FOXL2 and revealed that this factor interacts with proteins crucial for each developmental milestone of ovarian development. For example, we found that FOXL2 colocalizes with the transcription coactivator of Wnt signaling CTNNB1 (also known as β-catenin) throughout the time course. β-catenin activation was shown to induce male-to-female sex-reversal in a normal XY gonad (*93*), together with increased expression of FOXL2 and SOX9 down-regulation. The colocalization of β-catenin and FOXL2 may represent a mechanism to reinforce ovarian cell fate, suggesting that the functional interplay between the two pathways may rely on their joint recruitment to DNA. This was also supported by the identification of several other members of the Wnt pathway including USP7. USP7 is a disease-associated deubiquitinase (*94*), playing a role in chromatin remodeling through its histone deubiquitination activity (*79*, *80*), which can affect gene transcription activation and silencing (*95*). We found that USP7 is necessary for the differentiation and proliferation of the somatic cell lineages of both ovaries and testes. Given that *Usp7* mutant ovaries still express FOXL2, it is possible that USP7 may regulate granulosa cell differentiation and survival in a redundant way with FOXL2, as previously shown for other members of the Wnt signaling pathway (*85*), or, alternatively, that USP7 may regulate the function of other players involved in folliculogenesis, such as FOXL2 itself. It has recently been shown that USP7 can stabilize TF activity such as that of AR, by reversing polyubiquitination, thus allowing binding to DNA (*96*). We speculate that USP7 activity might affect FOXL2 recruitment to DNA, stabilizing this interaction and thus allowing the differentiation of granulosa cells in wild-type prepubertal ovaries. Because our ChIP-SICAP experiment allows for detection of proteins colocalizing on the same chromatin regions, it is also plausible that USP7 may not directly bind to FOXL2, but it could be enriched in the same chromatin regions, possibly contributing to gene regulation as shown in other systems, for example, mouse embryonic stem cells differentiation (*97*) and early adipogenesis (*98*). We noted that the *Usp7* gene was expressed throughout ovarian development to similar levels as of *Foxl2*. As we have seen with many other factors found to have a role in sex determination and/or gonadal sex identity such as WT1 (*55*), TRIM28 (*6*), CBX2 (*99*), and others (*100*), evaluating their role in these processes is challenging based solely on gene expression data, because they are often not sexually dimorphic, and their expression may not be restricted to the gonads. We believe that the power of our approach resides precisely in the ability to focus on proteins that are engaged on the DNA and, therefore, more likely to have an impact on gene regulation and cell differentiation and identity.

We have recently shown that the epigenetic regulator TRIM28 interacts with FOXL2 to maintain ovarian cell fate (*6*). TRIM28 also interacts with SOX9 in fetal testes (*101*). Our findings on USP7 add to the growing evidence supporting the role of epigenetic factors, often with bivalent roles in the two sexes, in the specification of somatic cell lineages of the gonads.

We identified four other interactors known to have a role in PFA that had been presumed to be FOXL2-independent, namely, NR5A2 (*58*), HDAC1 (*102*), HDAC6 (*59*), and casein kinase I isoform alpha (CSNK1A1) (*103*). This indicates that FOXL2 may create a chromatin hub where factors regulating similar processes can interact. Our study constitutes a precious resource for the discovery of novel regulators of PFA, a fundamental process that must be tightly regulated to ensure long lasting reproductive fitness. Recently, in vitro activation, a method for controlling PFA, has provided a possibility for conceiving to patients with POI (*104*). Identification of regulators of PFA may therefore also be invaluable to expand the toolset in reproductive technologies.

FOXL2 colocalized with WT1 throughout ovarian development. WT1 is a transcriptional regulator essential for early development of the gonads (*105*) existing in two main splicing isoforms, +KTS and −KTS. Only −KTS can act as a transcriptional regulator because the addition of the +KTS portion abrogates WT1 ability to bind to DNA, confining it to nuclear speckles (*106*, *107*). Given this knowledge and the fact that our FOXL2 ChIP-SICAP experiments specifically target proteins bound to chromatin, which is typically absent from nuclear speckles (*108*), our data support an interplay on the DNA of FOXL2 and WT1 −KTS, which may aid the suggested role of the latter in early ovarian development (*55*, *109*, *110*). We also identified interaction of FOXL2 with GATA4 (*57*, *111*). It was previously shown that deficiency in GATA4-FOG2 interaction impaired ovarian development through the alteration of the female gene expression program, including having a direct negative effect on *Foxl2* expression (*112*). Our data indicate that interaction with FOXL2 may be at the base of GATA4’s role in ovarian development. At 1W and 8W, we found FOXL2 interacting with ESR2, confirming that the previously postulated interaction between FOXL2 and estrogen signaling is mediated through the physical interaction of ESR2 and FOXL2 at proximal genomic locations (*39*, *113*). In addition, we detected AR at 1W and 8W as interactors, which support the motif analysis showing enrichment of AR motifs in FOXL2 binding sites. AR has a dual role, regulating granulosa cell proliferation in the ovary (*24*), while it is critical to ensure spermatogenesis is supported by Sertoli cells in the testis (*114*). This colocalization may hint to a role for AR in the transdifferentiation phenotype observed in the conditional *Foxl2* mutants (*7*).

We and others have found that FOXL2 physically interacts with several components of the splicing machinery. *Foxl2* knockdown was shown to induce missplicing in a series of genes (*52*), further supporting a role for FOXL2 in regulating this process. We found WTAP1, a Wilms’ tumor 1–associating protein with a role in splicing which is known to regulate cell cycle progression (*115*). Many other FOXL2 interactors were involved in DNA repair, adding to the growing body of evidence supporting a role of FOXL2 in directing double-strand breaks repair to protect cells from oxidative damage during cell cycle (*48*, *116*). Together, these data points toward a role for FOXL2 in other processes beyond transcription, including DNA repair and splicing, that are crucial to ensure granulosa cell survival and homeostasis throughout development and folliculogenesis.

We generated a *Foxl2*^*EGF*P^ mouse line to isolate FOXL2-positive cells. This was used to produce age-matched transcriptome and chromatin landscape datasets to integrate with our ChIP-SICAP data on the genomic binding sites. This integration allowed us to identify a high confidence set of genes likely to be repressed, activated, or dynamically regulated by FOXL2 across ovarian development. Our analysis showed that the majority of FOXL2 targets were either stably expressed throughout the time course or dynamic, while a lower number of genes were bound by FOXL2 and stably repressed. Among the stably expressed genes, we found genes with a role in mRNA processing, apoptosis, and cell cycle, functions that have all been linked with FOXL2 in functional studies (*116*). Analysis of those that are dynamically expressed and controlled by FOXL2 provided an overview of the range of functions critical for the ovary that this factor may regulate, from steroidogenesis to extracellular matrix organization. Genes involved in axon guidance were also highlighted, which is of interest as the ovary is innervated, whereas the testis is not (*117*). A smaller number of genes were stably repressed, including genes encoding for Sertoli structural proteins. We identified several potential enhancers important for granulosa cell differentiation, in genes such as *Rspo1* and *Runx1*. Particularly in the latter, several FOXL2 binding sites coincided with open chromatin regions, which supports the FOXL2-RUNX1 interplay in regulating ovarian cell fate maintenance (*8*). Last, the integration of ATAC-seq with FOXL2 ChIP-SICAP and comparison with published ATAC-seq datasets from sorted Sertoli cells (*50*, *118*) allowed us to observe that many genes important for Sertoli cells (e.g., *Amh*, *Cldn11*, *Itga6*, *Fgf9*, and *Hsd17b1*) contained regions open in both Sertoli and granulosa cells and that these were bound by FOXL2 in granulosa cells. This suggests that the role of FOXL2 in repressing the male pathway may go beyond only repressing *Sox9* and may unfold In addition through the direct repression of genes encoding proteins critical for the structure and function of Sertoli cells. It does not exclude, however, that it is the activation of *Sox9* and *Dmrt1*, the primary event leading to the gonadal sex reversal. The bivalent state of these regulatory regions also reveals that the system is permanently ready to undergo transdifferentiation from granulosa to Sertoli cells or vice versa. *Dmrt1* was not bound by FOXL2 in our study, suggesting the existence of other factors in granulosa cells that may be involved in its repression.

In summary, our study represents the first in vivo description of the chromatin interactome of the ovarian-specific TF FOXL2. We showed that this factor, essential for female fertility, differentially interacts with proteins playing a role in a range of gonadal-essential functions, from gonadogenesis, through follicular activation, to steroidogenesis and that this action is stage-specific. We identified a regulator of granulosa cell differentiation and proliferation, USP7, and showed that its deletion in the somatic cell component of the ovary leads to the inability of primordial follicles to exit the arrested pool stage and progress throughout folliculogenesis. In addition, we identified a defect in germ cell nest breakdown that suggests a role for USP7 in this process critical for primordial follicle formation (*119*).

In our study, we have applied chromatin proteomics to a notoriously challenging model, which is gonadal development, which often entails the handling of very low cell numbers, especially at embryonic stages. Our approaches could be optimized and further downscaled in the future, to be applied to even lower cell numbers. This would be especially valuable to query GRNs of rare cell populations of the gonads, such as the newly rediscovered Paired Box 8 (PAX8)–positive rete ovarii and rete testis (*120*). In addition, because, in ChIP-SICAP, an antibody is used to target a factor of interest, unless that factor is exclusively expressed in only one cell type within the organ targeted, then the experiment will likely yield interactors from multiple cell populations. While, in our case, we could assess the likely contribution of theca cells to our datasets, this may not always be possible in other systems. In that case, an additional isolation step to select for the population of interest before antibody pull-down for the factor of choice may be required. Given the current wide availability of single-cell transcriptomic datasets spanning gonadal development in both animal models and in humans (*70*, *87*, *121*, *122*), such markers could be easily identified in the future and harnessed to select specific cell populations before TF-targeted chromatin proteomics. We foresee that our approach could be used to gain more insights into the functional interactions shaping cell fate in gonadal development as well as in many other systems.

This study provides a rich resource of protein interactors colocalizing with FOXL2 on nearby genomic locations that could be used to generate hypotheses on the molecular mechanisms of ovarian development, POI, granulosa cell tumors, and other causes of female infertility.

## MATERIALS AND METHODS

### Animal procedures

All animal regulated procedures carried out were approved under the UK Animals (Scientific Procedures) Act 1986 and under the project licences 80/2405 and PP8826065 and Home Office guidelines and regulations. The *Foxl2^EGFP^* allele was maintained on a C57BL/6 background. *Foxl2^EGFP/+^* embryos were produced by crossing heterozygotes with C57BL/6 wild-type animals and collected at E14.5 after timed matings. Noon of the day of plug was considered as E0.5. Litters of 1-week-old postnatal mice were sexed, and females were culled to harvest the 1W time point. Fresh tissue was used for ATAC-seq and RNA-seq, while ovaries for ChIP-SICAP were snap-frozen and stored at −80°C until the day of the experiment. *Usp7^fl/fl^* mice were originally developed by Kon *et al.* (*83*) and were obtained from Novellasdemunt *et al.* (*123*). *Sf1:Cre^tg/+^*mice were developed by Bingham *et al.* (*84*).

### Generation of *Foxl2^EGFP^* reporter strain

The *Foxl2^EGFP^* reporter strain was generated by the Genetic Modification Service at the Francis Crick Institute using CRISPR-Cas9–assisted targeting to insert a P2A and EGFP sequence after Exon 1 in frame of the *Foxl2* sequence, followed by a stop codon (fig. S5, A to E). The donor vector contained a 783–base pair (bp) insert of P2A and eGFP, with 1-kb homology arms either side, synthesized by Thermo Fisher Scientific. The guide sequence used was 5′-TCTCTGAGTGCCAACGCGCG-3′ and was cloned into the Bbs I site of pX459 vector (Addgene, no. 62988). The gene targeting was performed by co-transfection of pX459 and the synthetic donor vector into B6N 6.0 embryonic stem cells using Lipofectamine 2000. Clones were screened using an insertion polymerase chain reaction (PCR) with primers within the EGFP (table S1), followed by a long-range PCR with primers across the entire transgene and Sanger sequencing. Copy number was evaluated using digital droplet PCR (ddPCR; fig. S5F), and one correctly targeted homozygous embryonic stem cells (ESC) clone was microinjected into goGermline blastocysts (*124*), which were then transferred into pseudo pregnant females. The resulting chimeras were crossed to C57BL/6J albino [B6(Cg)-Tyr^c-2J^/J] mice, and offspring were validated for the correct integration of the transgene using the above screening PCRs followed by Sanger sequencing confirmation of the resulting amplicons. The ddPCR was performed for copy number evaluation of the F1 heterozygous mice and no evidence of random integrations of the transgene was found. Long-read sequencing (Oxford Nanopore Technologies) was performed on two 3-kb amplicons spanning the targeted insertion, generated by PCR, and no aberrant on-target mutations or deletions were found 1.6 kb upstream and 1.8 kb downstream of the insertion.

### Immunofluorescence

Ovaries and testes were fixed after dissection by immersion in 4% paraformaldehyde at 4°C. Immunofluorescent stainings were performed on cryosections. The following primary antibodies were used: rabbit anti-SOX9 (Merck Millipore, catalog number AB5535), rat anti-GFP (Nacalai Tesque, 04404-84), goat anti-GFP (Abcam, ab5449), goat anti-FOXL2 (Abcam, ab5096), rabbit anti-FOXL2 (a gift from D. Wilhelm), rabbit anti-DDX4 (Abcam, ab27591), and rabbit anti-USP7 (Bethyl Laboratories, A300-033A). Slides were then incubated with Alexa Fluor secondary antibodies. Imaging of stained tissue sections was performed on a Leica SPE confocal microscope.

### High-resolution episcopic microscopy

*Usp7^fl/fl;^Sf1:Cre^tg/+^* mutant and *Usp7^fl/fl;^Sf1:Cre^+/+^* control ovaries were collected in phosphate-buffered saline (PBS) and immersed in Bouin’s fixative for a minimum of 12 hours at 4°C. Fixed tissue was extensively washed in PBS followed by dehydration in a series of graded methanol and 48 hours of incubation in a mix of JB-4/Eosine/Acridine orange to allow proper sample infiltration. Samples were embedded in fresh JB-4/Eosin/Acridine orange mix containing accelerator for polymerization (*125*, *126*). Embedded blocks were sectioned on a commercial oHREM (Indigo Scientific) at 0.70 μm, and sequential images of the block surface were acquired under GFP excitation wavelength light using Olympus MVX10 microscope and high-resolution camera (Jenoptik). The eosin gives the resin a fluorescent spectrum close to that of GFP, and it is the differential quenching of this depending on the nature of the tissue that gives a negative image of the sample. After acquisition, the stack was cropped to accommodate the sample volume and adjusted for gray level using Photoshop. Data were processed for isotropic scaling, orthogonal resectioning, and 25% downscaling with tailor made scripts. Three-dimensional volume rendering was produced using Osirix and/or Horos.

### Tissue collection and chromatin preparation for ChIP-SICAP

Ovaries from E14.5, 1W, and 8W C57BL/6 mice were dissected in PBS, snap-frozen, and stored at −80°C. A pool of 140 embryonic E14.5 gonads, 30 1W ovaries, and five 8W ovaries were used for each replicate. Chromatin was prepared using a modified version of the Active Motif High Sensitivity Chromatin Prep Kit protocol. Frozen tissue was pulverized under liquid nitrogen using mortar and pestle Bel-Art SP Scienceware Liquid Nitrogen-Cooled Mini Mortar. Ovaries were pooled to reach a minimum weight of 10 mg, allowing a chromatin yield of at least 10 μg per replicate. Samples were fixed in 1 ml of Fixation Buffer (1.5% methanol-free formaldehyde in 1% PBS) for 15 min on a roller at room temperature. Fixation was stopped using Stop buffer by Active Motif and incubating for 5 min. Chromatin preparation continued as per Active Motif protocol except for the sonication steps that were performed in a Bioruptor Plus sonication device by Diagenode with the following settings: 40 cycles of 30-s on/30-s off, “high” power, and constant temperature of 4°C.

### ChIP-SICAP and proteomics analysis

ChIP-SICAP experiments were performed in parallel using two biological replicates for each time point. ChIP-SICAP (*21*) allows the identification of chromatin-bound proteins that colocalize with a bait protein (FOXL2 in this study) on DNA. A total of 10 μg of sonicated chromatin was used for each immunoprecipitation using 3 μl of FOXL2 antibody donated by D. Wilhelm. We used a negative control (no antibody) in our ChIP-SICAP experiment to control for the background of the assay (that is to measure the proteins that are likely to be picked up by mass spectrometer in our samples even in the absence of antibody pull-down). This represent a mock-ChIP-SICAP, similarly to what is used for standard ChIP-qPCR and ChIP-seq experiments [see (*127*)]. As previously described (*21*), we used chromatin samples that we have taken through the protocol of ChIP-SICAP but omitting the antibody, as negative control. This way, we can most reliably measure all proteins that may be present in the assay but not bound by the FOXL2 antibody and subtract these from our analysis when measuring proteins which do bind to the FOXL2 antibody. To ensure specificity of our antibody, we have followed the Encyclopedia of DNA Elements (ENCODE)–recommended standards for ChIP (*86*) and validated our antibody with a primary characterization including immunofluorescence staining of wild-type adult ovaries, lacking FOXL2 [conditional deletion model as shown by Uhlenhaut *et al.* (*7*)], or adult testis. The original ChIP-SICAP protocol (*21*, *22*) was optimized to work on frozen tissue samples as follows. Chromatin was prepared with Active Motif chromatin preparation kit as described above. After overnight immunoprecipitation at 4°C with a FOXL2-specific antibody, immune-protein complexes were captured on protein G Dynabeads. DNA was biotinylated by terminal deoxynucleotidyl transferase in the presence of biotin-11-ddUTP and biotin-11-ddCTP as well as by Klenow3′exo in the presence of Biotin-7-dATP and eluted. Protein-DNA complexes were captured with protease-resistant streptavidin beads (*128*), and proteins were digested overnight at 37°C with 300 ng of LysC. Streptavidin beads carrying DNA were separated using a magnet to be processed for ChIP-seq library preparation. The supernatant, containing the digested peptides, was collected and digested further by 8 hours of incubation with 200 ng of trypsin. Digested peptides were cleaned using the stage-tipping technique. Briefly, digested samples were acidified by the addition of 2 μl of 10% trifluoroacetic acid (TFA). For each sample, 50 μl of 80% acetonitrile/0.1% formic acid was aliquoted in a LCMS Certified Clear Glass 12 mm–by–32 mm Screw Neck Total Recovery Vial (Waters). ZipTip with 0.6 μl of C18 resin was pre-treated by pipetting 100% acetonitrile twice by aspirating, discarding, and repeating. The ZipTip was equilibrated by pipetting 0.1% TFA, three times by aspirating, discarding, and repeating. Acidified samples were then pipetted 10 times with ZipTip to allow peptides to bind to the polymer within the tip. Following tip wash in 0.1% TFA, peptides were eluted in 80% acetonitrile/0.1% formic acid in the glass vial and the eluent dried in a speed vac. Peptides were reconstituted in 8 μl of 2% dimethyl sulfoxide/0.1% formic acid. Peptides were separated on a 50-cm, 75-μm–inside diameter Pepmap column over a 70-min gradient to be injected into the mass spectrometer (Orbitrap Fusion Lumos) according to the universal Thermo Fisher Scientific Higher-energy collisional dissociation (HCD) method. The instrument ran in data-dependent acquisition mode with the most abundant peptides selected for tandem mass spectrometry by HCD fragmentation. The raw data were analyzed using MaxQuant 2.0.1.0. The spectra were searched using the Swiss-Prot *Mus musculus* database. Variable modification included methionine oxidation and N-terminal acetylation. Fixed modification included cystenine carbamidomethylation. Trypsin and LysC were chosen as the enzymes, and maximum two missed cleavages were allowed. Peptide and protein false identification rate was set at false discovery rate (FDR) < 0.01. The quantification values were exported to R studio to analyze significantly enriched proteins using *t* test by limma package to determine Bayesian moderated *t* test *P* values and Benjamini-Hochberg (BH) adjusted *P* values and log_2_FC. We considered proteins with mean fold enrichment > 2 (log_2_FC > 1) and adjusted *P* < 0.1 as enriched proteins.

### DNA purification for ChIP-SICAP-seq samples

Protease-resistant streptavidin beads carrying DNA samples from the above ChIP-SICAP were processed for DNA purification. SDS buffer and proteinase K (20 mg/ml) were added to resuspend the beads followed by incubation for 30 min at 55°C. The beads were then heated at 80°C for 2 hours to reverse the cross-linking. A magnetic rack was used to separate the beads from the DNA, now in the supernatant. Supernatant was collected, and 50 μl of Ampure beads was used to purify the DNA. Purified input (no SICAP) and ChIP-SICAP DNA samples were used to generate libraries. A NEB Ultra II DNA kit was used. Samples were sequenced on the NovaSeq 6000 system. Paired-end sequencing was performed (30 million reads).

### Ovary dissociation for FACS

Ovaries were rinsed in 1× PBS and then dissociated using additional 450 μl of 0.05% trypsin and 50 μl of 2.5% collagenase. Tissue was incubated at 37°C for 6 min (for E14.5; up 20 min for 1W and 8W ovaries after mincing). Enzymatic reaction was quenched by adding 200 μl of PBS/3% fetal bovine serum (FBS). Supernatant was removed without disrupting the gonads; then, tissue clumps were resuspended in 300 μl of PBS/3% FBS and gently pipetted up and down until gonads were disaggregated. Cell mixture was passed through a 30-μm filter cap into a sorting tube. An extra 200 μl of PBS/3% FBS was used to rinse the tube and filtered. Samples were sorted with a FACSAria III to exclude debris (side scatter versus forward scatter) and doublets (area versus width) and to select only live cells by excluding cells positive for 4′,6-diamidino-2-phenylindole. Cells were collected directly into a low-retention 1.5-ml tube with 200 μl of PBS/3% FBS and kept on ice.

### RNA extraction, library preparation, and sequencing

A total of 50,000 GFP-positive cells were sorted, and RNA was extracted using the RNeasy Plus Micro Kit (catalog number 74034) by Qiagen. RNA quality was assessed by Bioanalyzer, and samples with a RNA integrity number (RIN) value > 8 were processed for library preparation. A total of 10 ng of RNA was used to prepare libraries using the NEBNext Low Input RNA Library Prep Kit (E6420L). Samples were subsequently sequenced on the NovaSeq 6000 system, at a depth of 25 million reads (SR75) per sample.

### ATAC-seq, library preparation, and sequencing

GFP-positive cells isolated by FACS from gonads at E14.5, 1W, and 8W (three biological replicates per time point) were used for ATAC-seq library preparation using the Active Motif ATAC-Seq Kit (catalog number 53150). In brief, 100,000 GFP-positive sorted cells were washed in PBS, pelleted, and lyzed in the ATAC Lysis Buffer to isolate intact nuclei. Tagmentation of nuclei was performed for 30 min in a thermomixer at 37°C set at 800 rpm. Tagmented DNA was purified using DNA purification columns, and library amplification was performed using Illumina’s Nextera adapters. A unique combination of i7/i5 primers was used to allow for multiplexing and sequencing on the same flow cell. PCR amplification included 5 min of incubation at 72°C, followed by 30 s of DNA denaturation at 98°C and 10 cycles of 98°C for 10 s, 63°C for 30 s, and 72°C for 1 min. PCR products were cleaned up using SPRI beads, and size distribution of PCR-amplified libraries was assessed on TapeStation. Samples were subsequently sequenced on the NovaSeq 6000 system.

### mRNA-seq alignment and quantification

Reads were processed using the publicly available nf-core (*129*) rnseq pipeline v3.3 with the STAR (*130*)/RSEM (*131*) option against mouse genome assembly GRCm38 and Ensembl release 81 transcript annotations. Other options were left as default. Gene-level RSEM abundance estimates were imported into R using the Bioconductor package tximport’s tximport function (*132*) for further analysis using DESeq2 (*71*). Normalization factors were calculated per gene using the default DESeq2 function, correcting for library size and feature length. VST abundance estimates were calculated using the VST function. PCA analysis was performed on the VST values using all available genes.

### mRNA-seq differential expression analysis and clustering

Differential expression between treatment groups was assessed using a DESeq2’s Wald test in a pairwise manner, with FDR control based on an independent hypothesis weighting (*133*). Shrunken log_2_FCs were calculated using the lfcShrink function (type = “ashr”). Significance was assessed on the basis of a combined FDR < 0.01, a shrunken log_2_FC > 2, and a *baseMean* > 2 (i.e., mean abundance across all samples). A combined list of significantly DEGs from all pairwise comparisons was created and used to generate a heatmap showing how patterns of expression changed across samples. Per-gene *z*-scores were calculated from the VST abundance estimates. *K*-means clustering was applied (*km* = 7) to split the data into slices of similarly changing genes. Within each slice, genes were clustered using a Euclidean distance metric and complete linkage.

### GO enrichment analysis

GO BP (BP enrichment analysis of the individual gene lists and the *k*-means clusters) was calculated relative to a genomic background via the compareCluster function from the clusterProfiler Bioconductor package (*134*) (fun = “enrichGO,” OrgDb = “org.Mm.eg.db,” ont = “BP,” pAdjustMethod = “BH,”* pvalueCutoff = 0.05*, *qvalueCutoff = 0.01*, *minGSSize = 10*, *maxGSSize = 500*).

### ChIP-SICAP: ChIP-seq read alignment, peak calling, and peak annotation

Reads were processed using the publicly available nf-core (*129*) chipseq pipeline v1.2.2 against the mouse genome assembly GRCm38 (--narrow_peak, --single_end, --deseq2_vst, min_reps_consensus 2, --macs_fdr 0.05), applying the Encode GRCm38 blacklist definitions to filter out problematic regions. A peak was only considered to be genuine if present in both biological replicates for each treatment group. A consensus peak set was then constructed by combining the peak calls from the individual treatment groups into a set of nonoverlapping intervals. Consensus peaks were annotated using the Bioconductor package ChIPpeakAnno’s annotatePeakInBatch function (*135*) against the protein-coding gene component of Ensembl release 81. Gene assignments were made on the basis of peak-center minimal proximity to a gene’s transcription start site (TSS) (output = “nearestLocation,” multiple = FALSE, maxgap = 1 L, PeakLocForDistance = “middle,” FeatureLocForDistance = “TSS,” select = “first,” bindingType = “fullRange”). The distribution of peaks mapping to genomic features was similarly assessed using ChIPpeakAnno’s “genomicElementDistribution” function, defining promoter regions as ±2 kb around a TSS and downstream regions as the 2-kb region downstream of genic boundaries.

### DiffBind analysis

Consensus peak occupancy analysis was conducted using the Bioconductor package DiffBind (*136*). Briefly, reads were counted from the nfcore chipseq pipeline alignment files Binary Alignment Map (BAM) over a combined set of consensus peaks (intervals). Control (input) read counts were subtracted from the targeted samples at each interval: dba.count (samples.dba, peaks = consensus.gr, summits = FALSE, filter = 0, bSubControl = TRUE, minCount = 0, bUseSummarizeOverlaps = TRUE, mapQCth = 0). The resulting DiffBind object was normalized using a background approach, which calculates scaled factors from read counts against a set of 15,000-bp genomic bins considered large enough not to show differential enrichment across samples: dba.normalize (samples.dba, background = TRUE, method = DBA_DESEQ2). Normalized peak counts for all peaks were subsequently used for Pearson correlation analysis and PCA analysis. Differential binding affinity analysis was conducted in a pairwise fashion between treatment conditions using DESeq2 (*71*). An FDR of <0.05 was used to threshold significant changes in peak occupancy. Peaks showing significant changes in occupancy were submitted for read-depth profiling in the region ±1.5 kb from their centers. Plots are presented at the merged replicate level and stratified on the basis of gain/loss status (i.e., direction of fold change).

### Clustering of ChIP-seq peaks

The normalized peak occupancy estimates were used to cluster the consensus peaks into sets that showed similar patterns of variation in occupancy across conditions. Peak estimates were merged across replicates using a mean, a pseudo-count of 0.1 was added before a log_2_ transformation. Per-peak z-scores were calculated. *K*-means clustering was applied (*km* = 7, 1000 repeats) to split the data into slices of similarly behaving peaks. Within each slice peaks were clustered using a Euclidean distance metric and complete linkage.

### Motif enrichment analysis

Analysis was performed using the “calcBinnedMotifEnrR” from the monaLisa package (*74*), by scanning the sequence of consensus peaks ±500 bp from their centers against the PWMs from JASPAR 2020 (*137*). Significance of enrichment was assessed against a background of equivalently sized sequences randomly sampled from the genome. Sequences were weighted to correct for guanine-cytosine (GC) content and *k*-mer composition differences between fore- and background sets. Statistically significant enriched motifs within each cluster were determined using a one-tailed Fisher’s exact test with BH adjusted *P* values < 0.0001 and subsequently prioritized on the basis of their log_2_ enrichment scores (descending).

### ATAC-seq

#### Read alignment and peak calling

Reads were processed using the publicly available nf-core (*129*) atacseq pipeline v1.2.1 against the mouse genome assembly GRCm38 (--deseq2_vst --min_reps_consensus), applying the Encode GRCm38 blacklist definitions to filter out problematic regions. A peak was only considered to be genuine if present in at least two biological replicates for each treatment group. A consensus peak set was then constructed by combining the peak calls from the individual treatment groups into a set of nonoverlapping intervals.

#### Peak annotation

Consensus peaks were annotated using the Bioconductor package ChIPpeakAnno’s annotatePeakInBatch function (*135*) against the protein-coding gene component of Ensembl release 81. Gene assignments were made on the basis of peak-center minimal proximity to a gene’s TSS (output = “nearestLocation,” multiple = FALSE, maxgap = −1 L, PeakLocForDistance = “middle,” FeatureLocForDistance = “TSS,” select = “first,” bindingType = “fullRange”). The distribution of peaks mapping to genomic features was similarly assessed using ChIPpeakAnno’s genomicElementDistribution function, defining promoter regions as ±2 kb around a TSS and downstream regions as the 2-kb region downstream of genic boundaries.

#### DiffBind analysis

Consensus peak occupancy analysis was conducted using the Bioconductor package DiffBind (*136*). Briefly, reads were counted from the nfcore chipseq pipeline alignment files (BAM) over a combined set of consensus peaks (intervals) dba.count (samples.dba, peaks = consensus.gr, summits = FALSE, filter = 0, bSubControl = FALSE, minCount = 0, bUseSummarizeOverlaps = TRUE, mapQCth = 0). The resulting DiffBind object was normalized DESeq2’s RLE method (*71*) on the basis of the peak counts dba.normalize (samples.dba, method = DBA_DESEQ2, normalize = DBA_NORM_RLE, library = DBA_LIBSIZE_PEAKREADS). Normalized peak counts for all peaks were subsequently used for Pearson correlation analysis and PCA analysis.

Differential binding affinity analysis was conducted in a pairwise fashion between treatment conditions using DESeq2 (*71*). An FDR of <0.05 was used to threshold significant changes in peak occupancy. Peaks showing significant changes in occupancy were submitted for read-depth profiling in the region ±1.5 kb from their centers. Plots are presented at the merged replicate level and stratified on the basis of gain/loss status (i.e., direction of fold change).

#### Clustering of peaks

The normalized peak occupancy estimates were used to cluster the consensus peaks into sets that showed similar patterns of variation in occupancy across conditions. Peak estimates were merged across replicates using a mean, a pseudo-count of 0.1 was added before a log_2_ transformation. Per-peak *z*-scores were calculated. *K*-means clustering was applied (*km* = 7, 1000 repeats) to split the data into slices of similarly behaving peaks. Within each slice peaks were clustered using a Euclidean distance metric and complete linkage.

#### Data integration

Data from the RNA, ATAC, and ChIP experiments were integrated at the gene level. The ATAC and ChIP data were assigned to protein-coding genes based on minimal peak proximity to a TSS. Where this resulted in multiple peaks being assigned the same gene, the one closest to the TSS was given precedence. Identification of stably expressed and dynamically regulated genes: genes dynamically regulated by FOXL2 were defined as the intersect of (i) genes differentially expressed in at least one of the pairwise comparisons from the RNA-seq analysis and (ii) bound by Foxl2 based on the presence of a ChIP-seq peak in at least one time point. Stably expressed genes were defined as those bound by FOXL2 but now showing differential expression. Genes not bound by FOXL2 but showing differential expression were not considered to be directly regulated by FOXL2. Stably expressed genes were subsequently divided into “activated” and “repressed” subsets on the basis of the VST normalized counts from the RNA-seq analysis. A VST score of ≥8.3 in at least one sample was enough to quality a gene as activated, while all others were regarded as repressed.

#### Cytoscape visualization of FOXL2 protein interactors

GO enrichment analysis of FOXL2 interactors identified by ChIP-SICAP and fold changes over no-antibody control in table S3 were loaded onto Cytoscape v.3.9.1 (*138*). Proteins were clustered on the basis of their function. The cytoscape app enhancedGraphics (*139*) was used to overlay bar charts depicting the fold changes over control of each protein in each time point.
