# Supplementary material for: FOXL2 interaction with different binding partners regulates the dynamics of ovarian development
Source: Sci Adv. 2024 Mar 22;10(12):eadl0788. doi: 10.1126/sciadv.adl0788 (PMC10959415; doi:10.1126/sciadv.adl0788)
Supplement: Supplementary file 1 — Figs. S1 to S16 Table S1 Legend for movie S1 Legends for data S1 to S6 [file sciadv.adl0788_sm.pdf]

Science Advances
AAAS

Supplementary Materials for

FOXL2 interaction with different binding partners regulates the dynamics of ovarian development

Roberta Migale *et al.*

Corresponding author: Roberta Migale, roberta.migale@crick.ac.uk; Robin Lovell-Badge, robin.lovell.badge@crick.ac.uk

Sci. Adv. **10**, eadl0788 (2024)
DOI: 10.1126/sciadv.adl0788

The PDF file includes:

Figs. S1 to S16
Table S1
Legend for movie S1
Legends for data S1 to S6

Other Supplementary Material for this manuscript includes the following:

Movie S1
Data S1 to S6

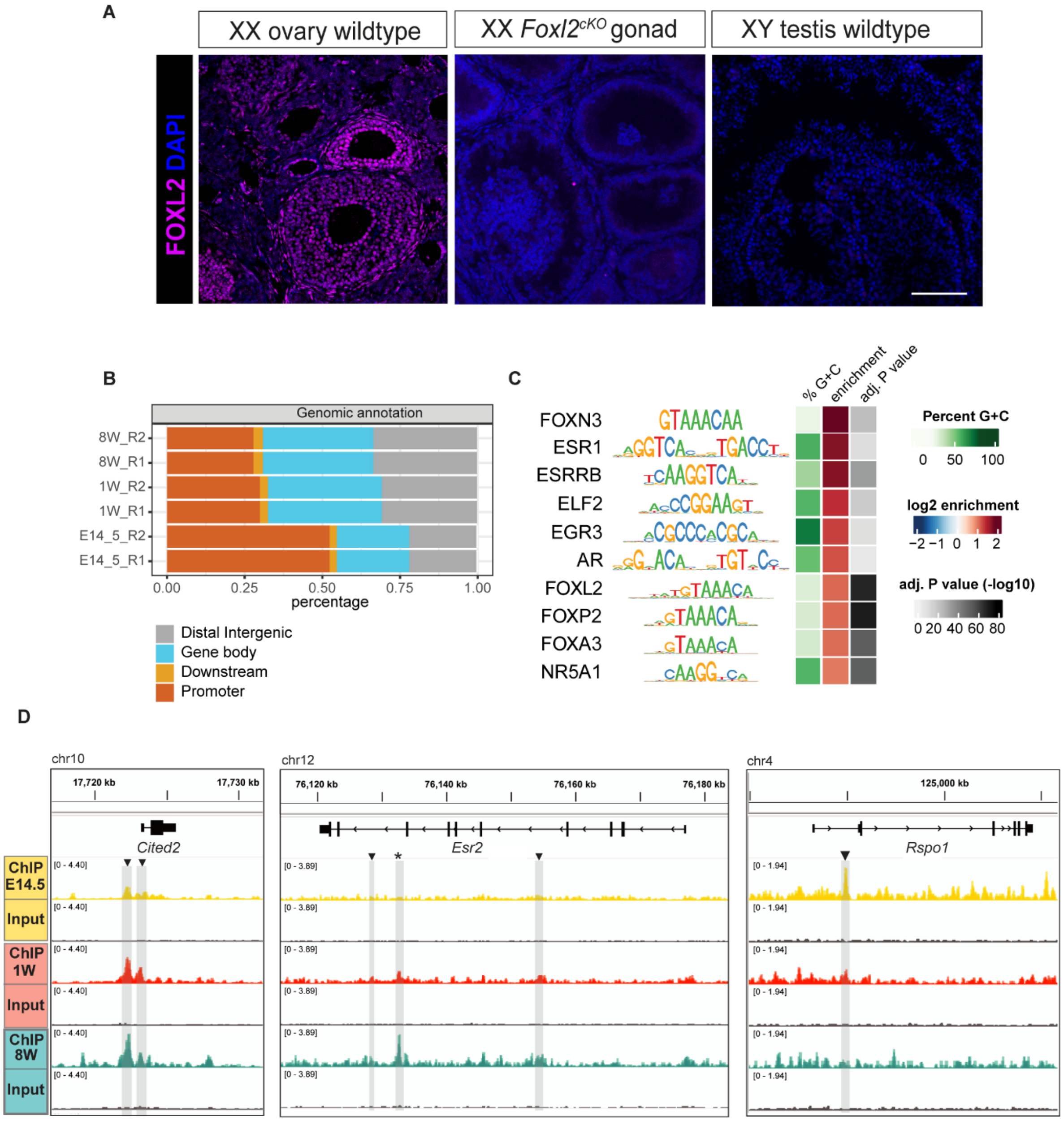

Figure S1. FOXL2 genome-wide occupancy across ovarian development.

(**A**) Immunofluorescence analysis of FOXL2 expression confirms the specificity of the antibody used for ChIP-SICAP. Cryosections of wildtype ovaries, FOXL2 conditional knockout ovaries, as shown by Uhlenhaut *et al.* (*7*), and wildtype testes. Scale bar = 100µM. (**B**) Annotation of consensus peaks by ChIPpeakAnno. (**C**) Enrichment analysis of transcription factor binding sites associated with consensus peaks significantly changing across the timecourse. Analysis performed using monaLisa package (*74*). Top 10 representative TF are shown (*p-value*<0.0001). (**D-F**) IGV tracks representative of FOXL2-bound regions. Main tracks show normalised read-depth coverage. Grey bars and black arrows highlight significant peaks over input. Asterisk denotes a previously identified functional enhancer of *Esr2* (E) (*39*).

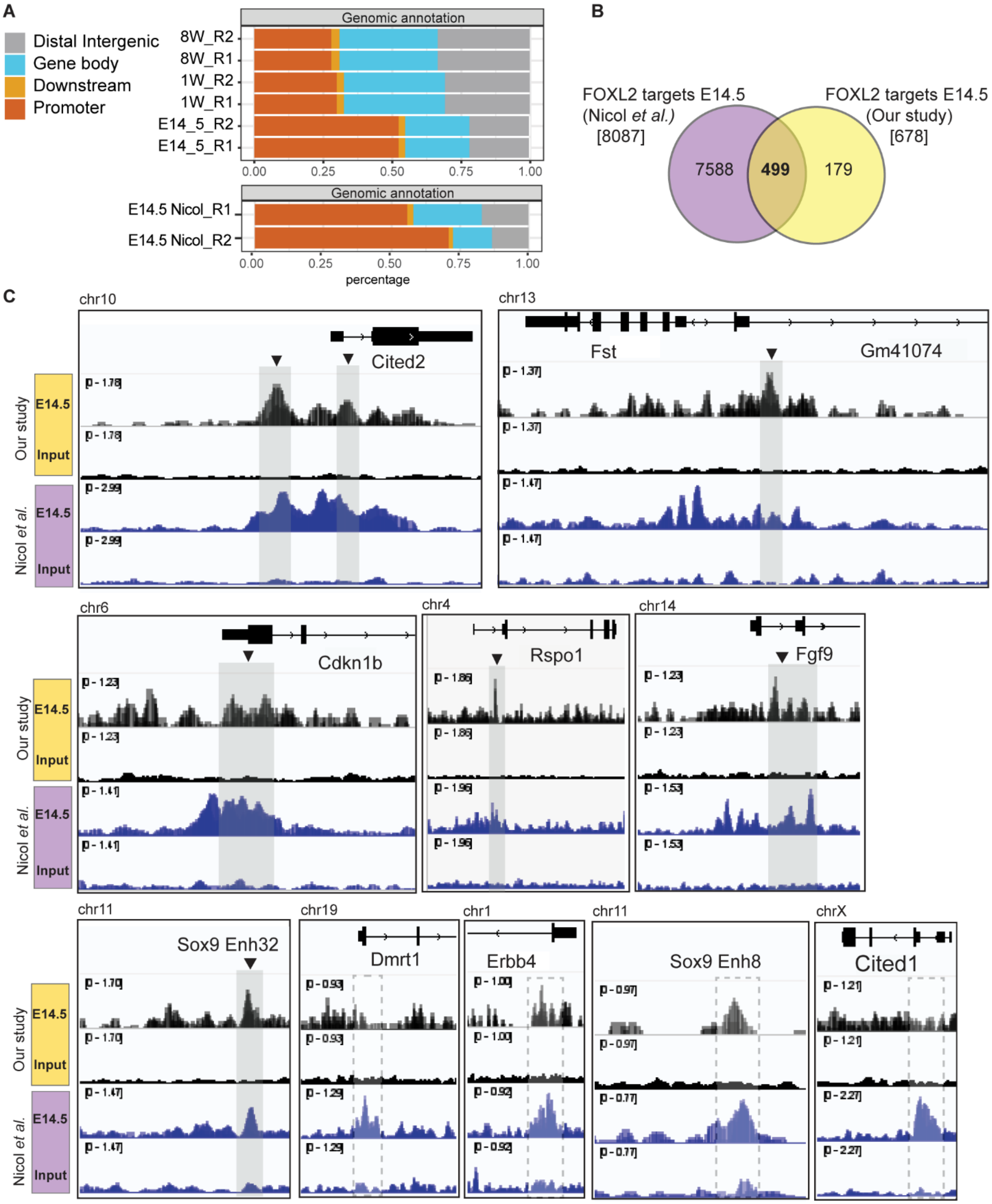

Figure S2. Comparison of our E14.5 ChIP-SICAP timepoint with a published ChIP-Seq dataset of FOXL2 ChIP-Seq by Nicol *et al.* (*29*) (**A**) Annotation of consensus peaks by ChIPpeakAnno. (**B**) Overlap between the peaks identified by our studies. (**C**) IGV tracks of genomic binding of FOXL2 peaks in representative genes. Grey boxes represent peaks identified as significant in our study. Dotted boxes indicate peaks not significant in our study but significant in (*29*).

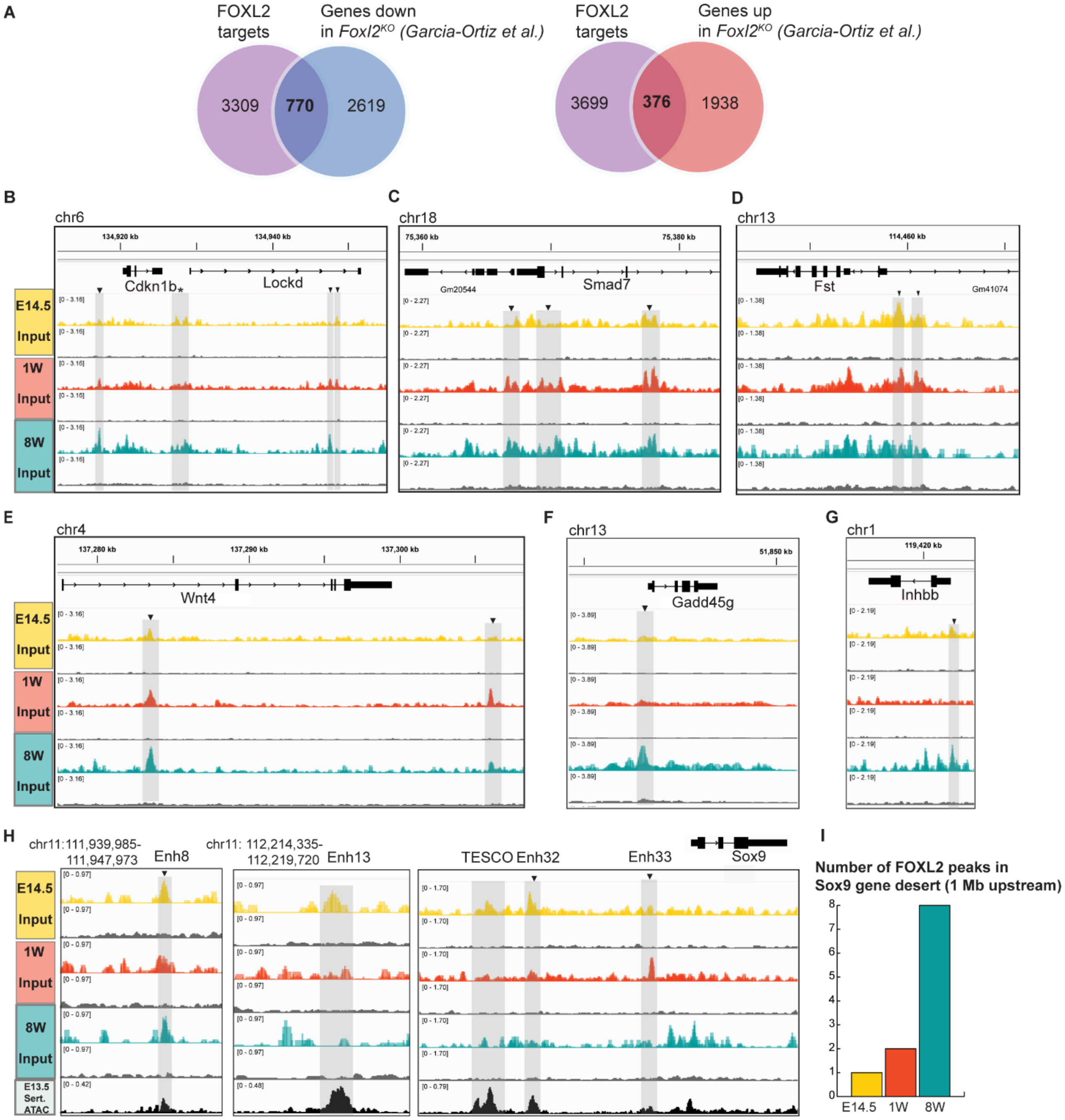

Figure S3. FOXL2 genome-wide occupancy across ovarian development.

(**A**) Venn diagram showing the overlap between FOXL2 target genes identified at any point throughout ovarian development (E14.5, 1W and 8W) and total of genes downregulated/upregulated in *Foxl2*$^{-/-}$ null mutant ovaries collected at 13.5, 16.5 dpc and birth compared to wildtype controls ((*28*) and Data S2). Genomic overviews of FOXL2 peaks in representative genes likely to be activated by FOXL2 including *Cdkn1b* (**B**), *Smad7* (**C**), and *Fst* (**D**). Representative genes potentially downregulated by FOXL2, including *Wnt4* (**E**), *Gadd45g* (**F**), *Inhbb* (**G**) and *Sox9* (**H**). (I) Bar chart displaying the total number of significant peaks identified in each timepoint in the 1Mb gene desert upstream of *Sox9*.

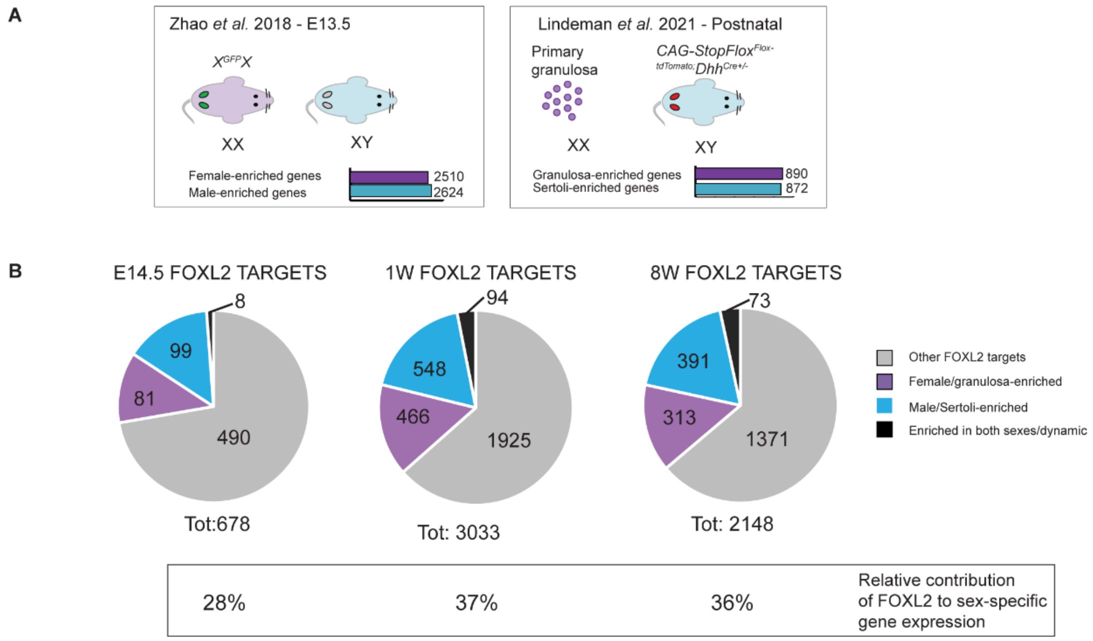

Figure S4. The contribution of FOXL2 to the regulation of granulosa and Sertoli-enriched genes is greater at postnatal stages.

(**A**) Overview of datasets used: RNA-Seq analysis performed on XX and XY E13.5 gonads, from (*49*), and RNA-Seq analysis from *(50)* of primary granulosa cells isolated from 23-29 days old mice, compared to Sertoli cells from P7 XY pups. Bar charts indicate the number of genes enriched in either ovary/granulosa (purple) or testis/Sertoli cells (blue). (**B**) Pie charts depicting the proportion of FOXL2 target genes, as identified at E14.5, 1W or 8W, and classified as either ovary/granulosa- or testis/Sertoli. Grey denotes other FOXL2 target genes identified by our ChIP-SICAP and not overlapping with the lists of granulosa/Sertoli-enriched genes from the two studies.

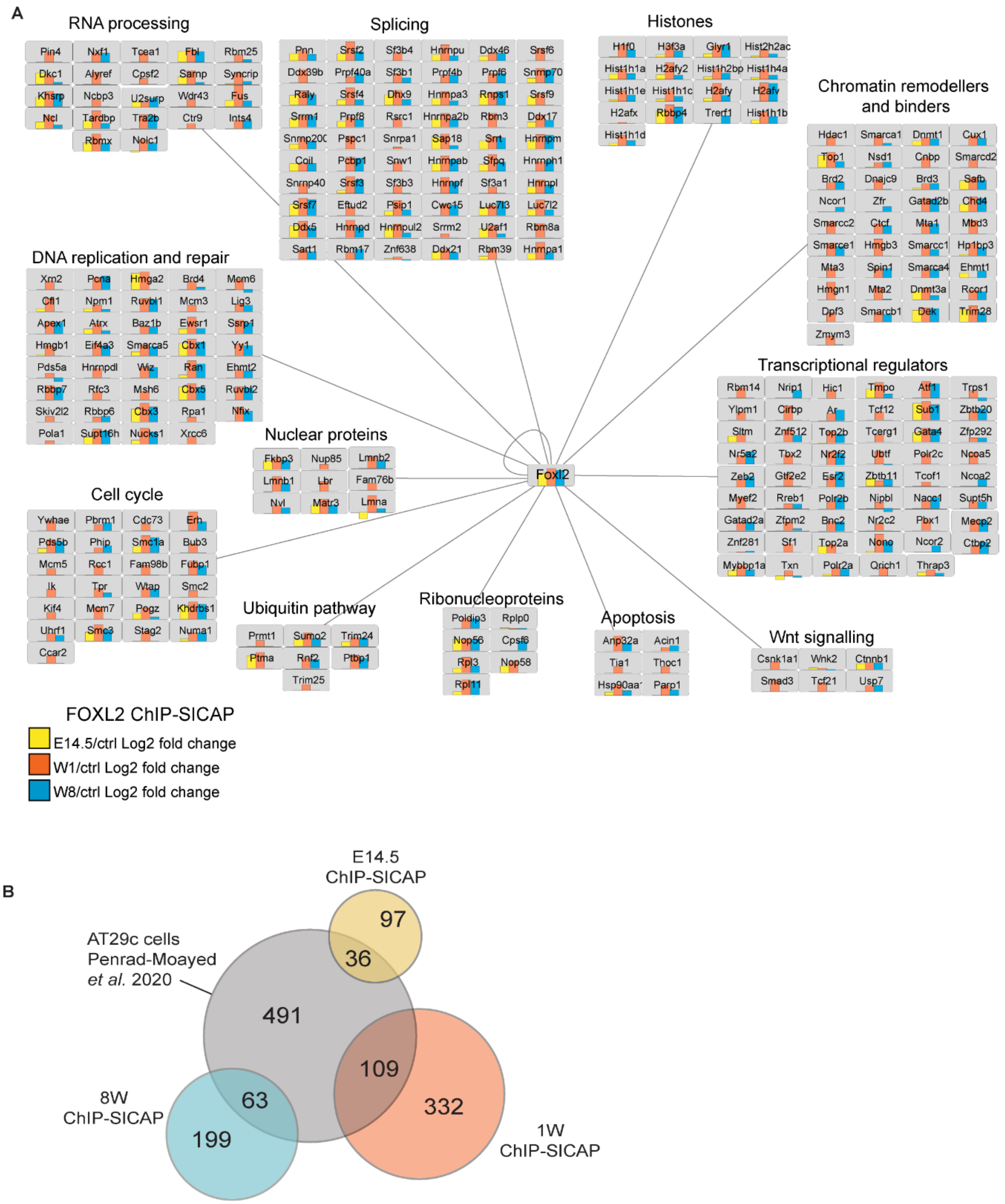

Figure S5. Cytoscape network of FOXL2 interactome across ovarian development.
(**A**) Proteins interacting with FOXL2 on-chromatin were clustered by GO Processes and visualised with Cytoscape. Enrichment values over no-antibody control were depicted as barcharts (yellow=E14.5, orange=1W, blue=8W, n=2, fold change over no antibody control >2 in at least one timepoint, adj-*pvalue*<0.1) and visualised within the network using enhancedGraphics Cytoscape plugin (*119*). (**B**) Overlap of protein interactors found in our study compared to a cell line (AT29c) whole proteome study by Penrad-Mobayed *et al.*(*52*).

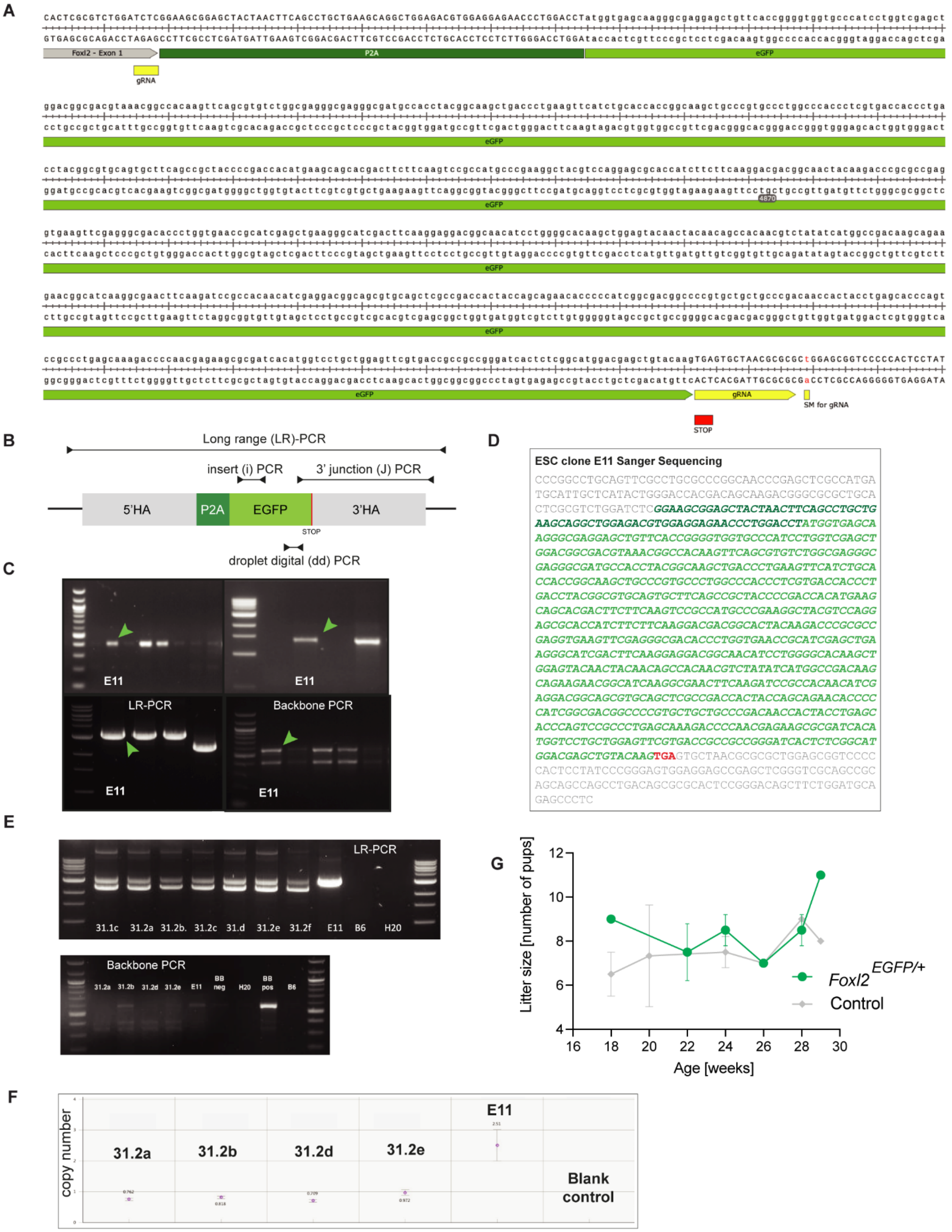

Figure S6. Design of *Foxl2^{EGFP}* construct, genotyping strategy, and fertility assessment of the *Foxl2^{EGFP}* mouse line.

(**A**) Design of gRNA (yellow box) and associated silent mutation (SM) in PAM sequence as well as 783bp P2A-EGFP construct (green boxes). (**B**) Location of primers for insert, junction, long-range and digital droplet PCR. (**C**) ES cell screening identified clone E11 (green arrows), which showed successful EGFP integration depicted by a single band in insert, junction, and long-range PCR. (**D**) Correct integration of EGFP into Foxl2 locus of clone E11 was confirmed via sanger sequencing. (**E**) Long-range PCR of chimeric offspring as well as clone E11 confirmed heterozygous and homozygous EGFP insertion, respectively. No evidence of backbone integration was found. BB pos= backbone positive; BB nrg= backbone negative. (**F**) Copy number evaluation of chimeric offspring as well as ES cell clone E11 by ddPCR confirmed absence of random transgene integration. (**G**) Fertility assessment of $Foxl2^{EGFP/+}$ females compared to wildtype controls (C57BL/6J stock mice).

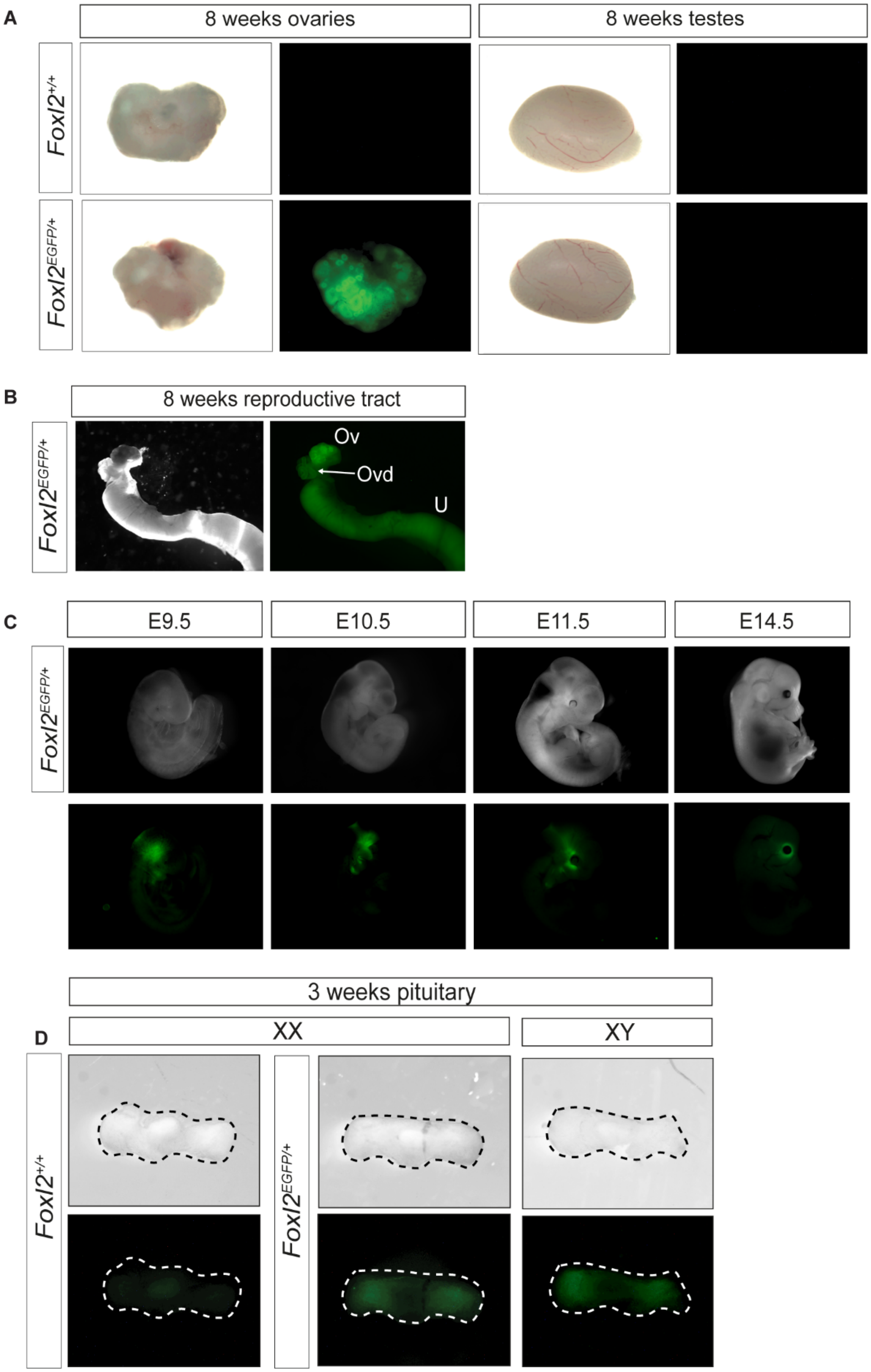

Figure S7. Characterization of the *Foxl2^EGFP^* mouse line. Detection of endogenous EGFP in freshly collected tissues expressing FOXL2. (**A**) Representative images of ovaries and testes dissected from adult mice (8 weeks) and imaged with a fluorescence microscope. Left: brightfield channel, right: GFP channel. Adult mouse ovaries wildtype, heterozygous, as well as testes

wildtype and heterozygous are shown. (**B**) Section of adult female reproductive tract including uterus, oviduct and ovary showing EGFP expression in the ovary and uterus. (**C**) EGFP fluorescence in developing mouse embryo (XX). **(D**) 3-weeks old pituitary glands showing EGFP fluorescence in XX and XY *Foxl2*$^{EGFP/+}$ mice. *Foxl2*$^{+/+}$mice were collected as controls and show no EGFP expression.

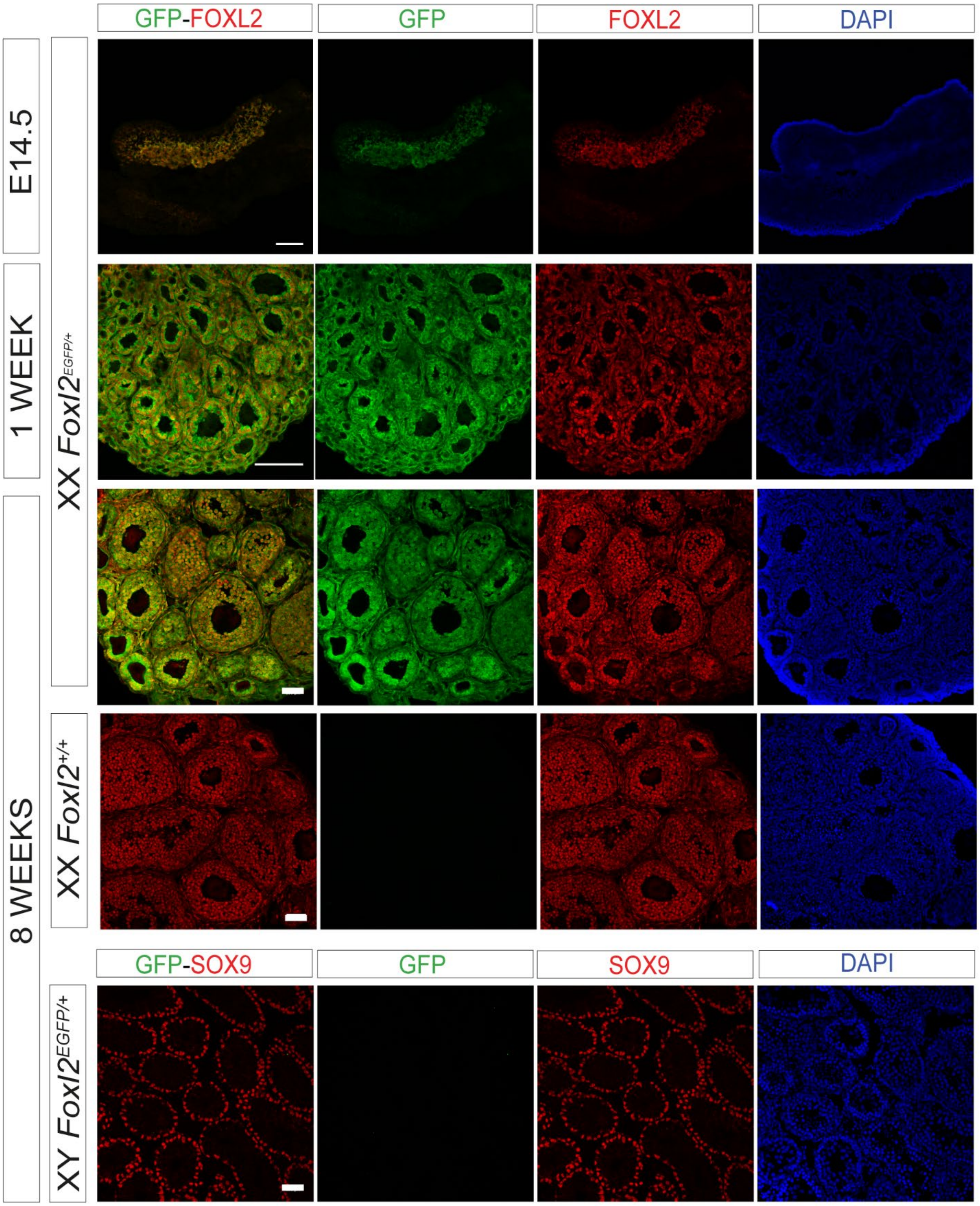

Figure S8. Characterization of the *Foxl2*^{EGFP} mouse line by immunofluorescence. Immunofluorescence staining of EGFP (green) and endogenous FOXL2 (red) in E14.5, 1 week and 8-weeks old XX *Foxl2*$^{EGFP/+}$ ovaries, and of EGFP and endogenous SOX9 (red) in XY *Foxl2*$^{EGFP/+}$ adult testes. Scale bars: 100 µm.

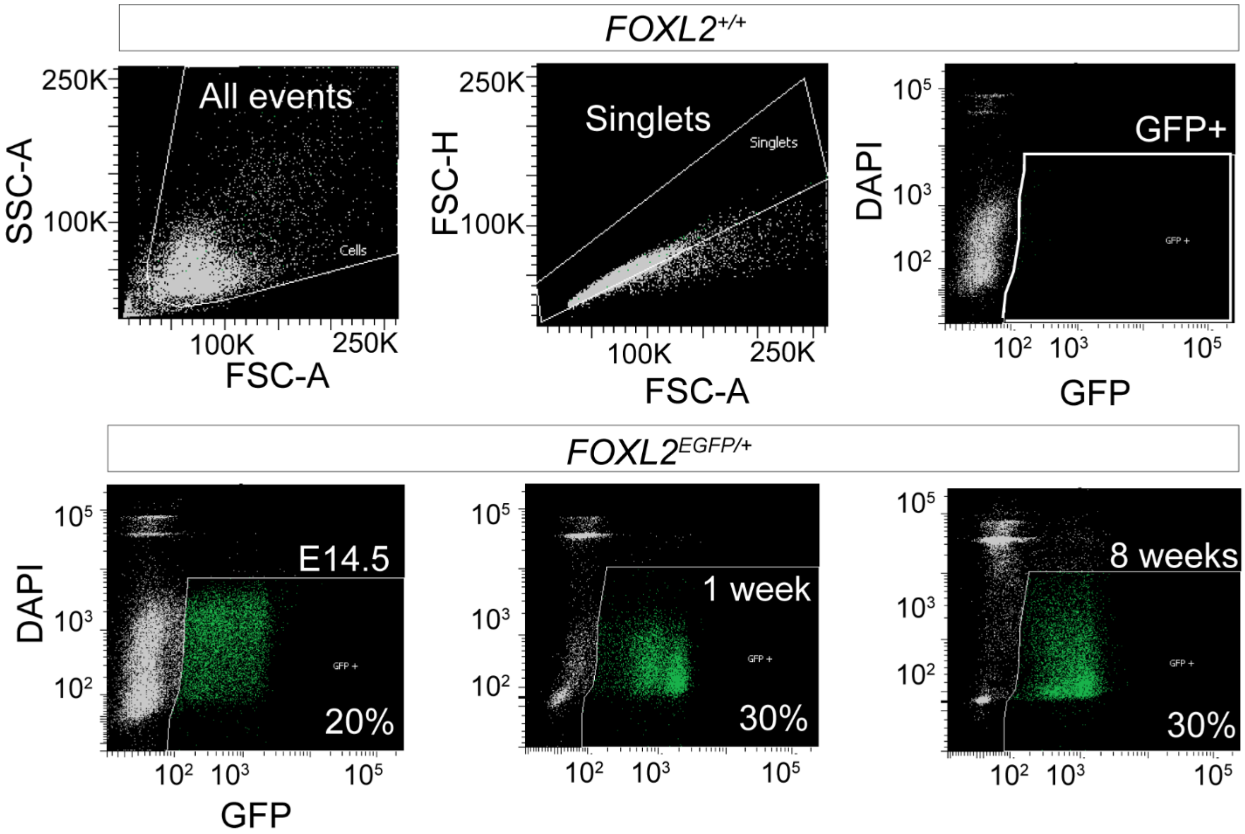

Fig. S9. Gating strategy to isolate *Foxl2*$^{EGFP/+}$ cells by FACS.

Fluorescence-activated cell sorting of *Foxl2*$^{EGFP/+}$ cells positive cells throughout ovarian development. Overview of gating strategy used to isolate live cells, singlets, and positive for EGFP from *Foxl2*$^{EGFP/+}$ ovaries. An example of FACS profile of a negative sample from *Foxl2*$^{+/+}$ ovaries is shown and it was used to set the gates.

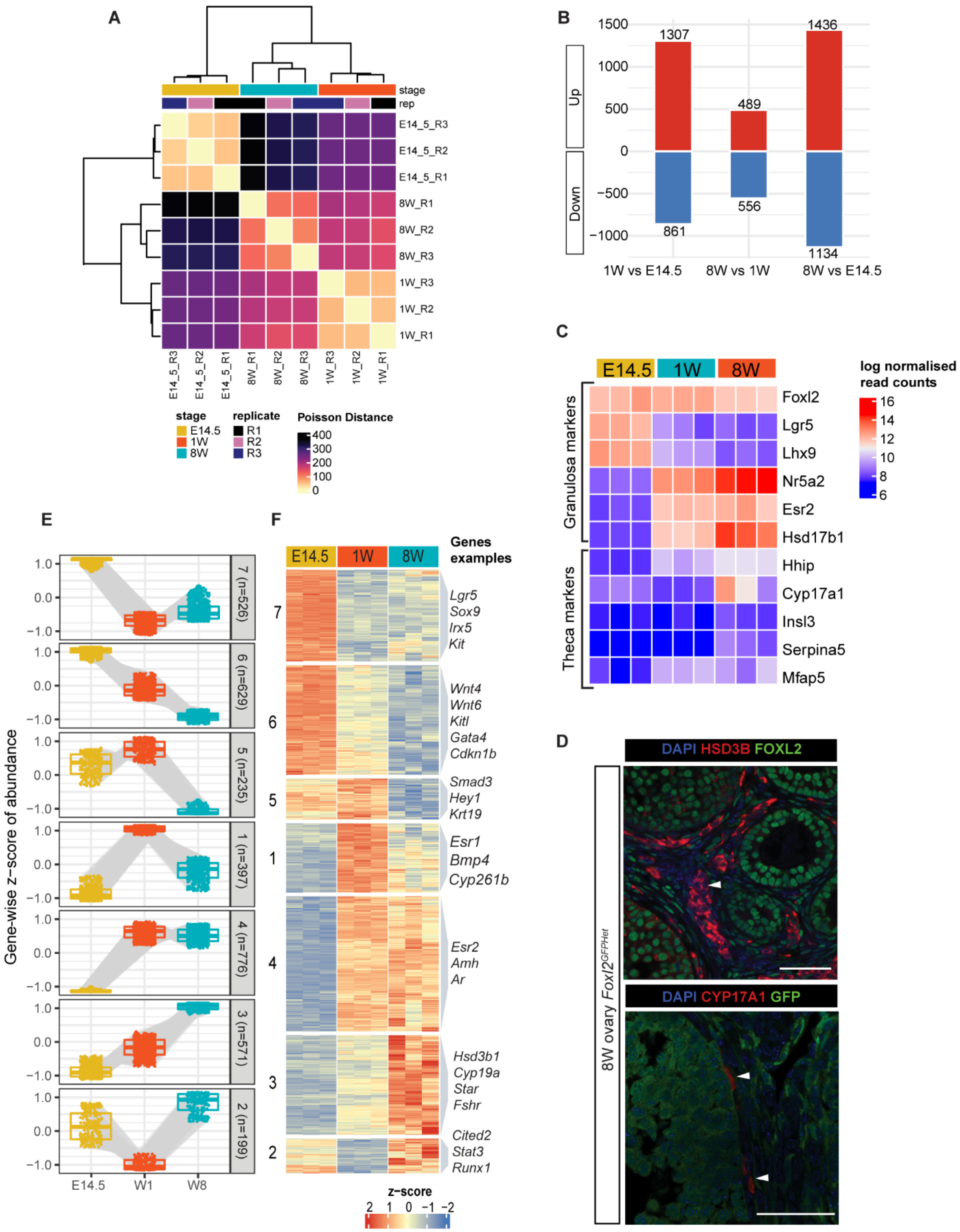

Fig. S10. Overview of RNA-Seq analysis of *Foxl2*$^{EGFP/+}$ cells isolated throughout ovarian development. (**A**)Poisson dissimilarity metric to assess RNA-Seq sample similarity, n=3 biological replicates. (**B**) Bar plot summary illustrating the number of DEGs identified in each pairwise comparison (*pvalue*<0.01, fold-change >2). (**C**) Heatmap of gene expression changes of

granulosa/theca cell markers. Log normalized read counts are shown. (**D**) Immunofluorescence analysis of expression markers of theca (HSD3ß, CYP17A1), and granulosa (GFP, FOXL2) cells in adult mouse ovary sections. (**E**) Boxplot illustrating the dynamics of gene expression changes granulosa *Foxl2*$^{EGFP/+}$ cells. (**F**) Hierarchical k-means clustering of the gene expression dynamics and representative genes on the right-hand side.

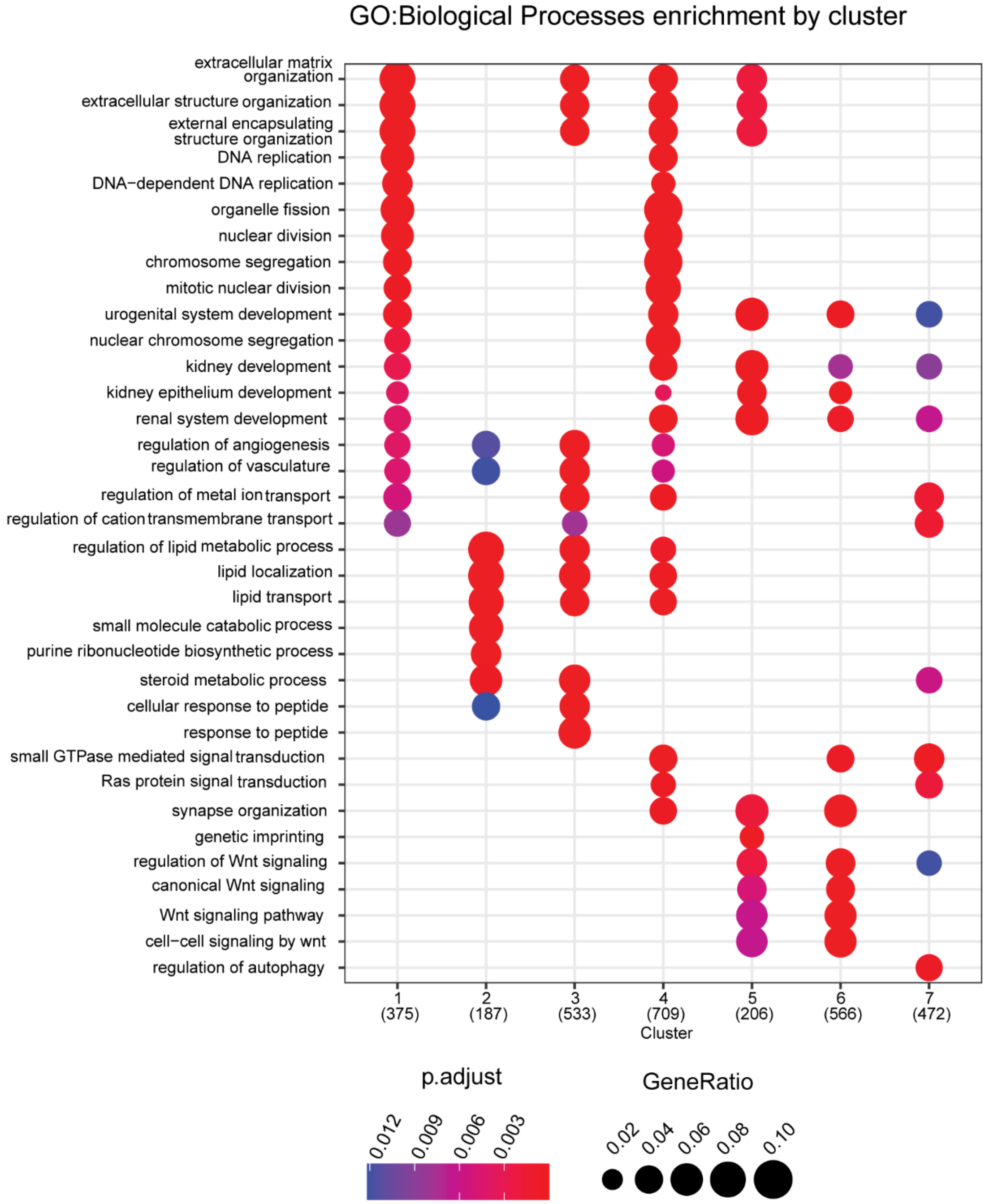

Fig. S11. Gene Ontology Biological Processes Enrichment analysis of genes differentially expressed across ovarian development in *Foxl2*$^{EGFP/+}$ mouse ovaries. Top terms from the GO enrichment test (Cluster Profiler) showing the processes associated with DEGs from each of the seven clusters.

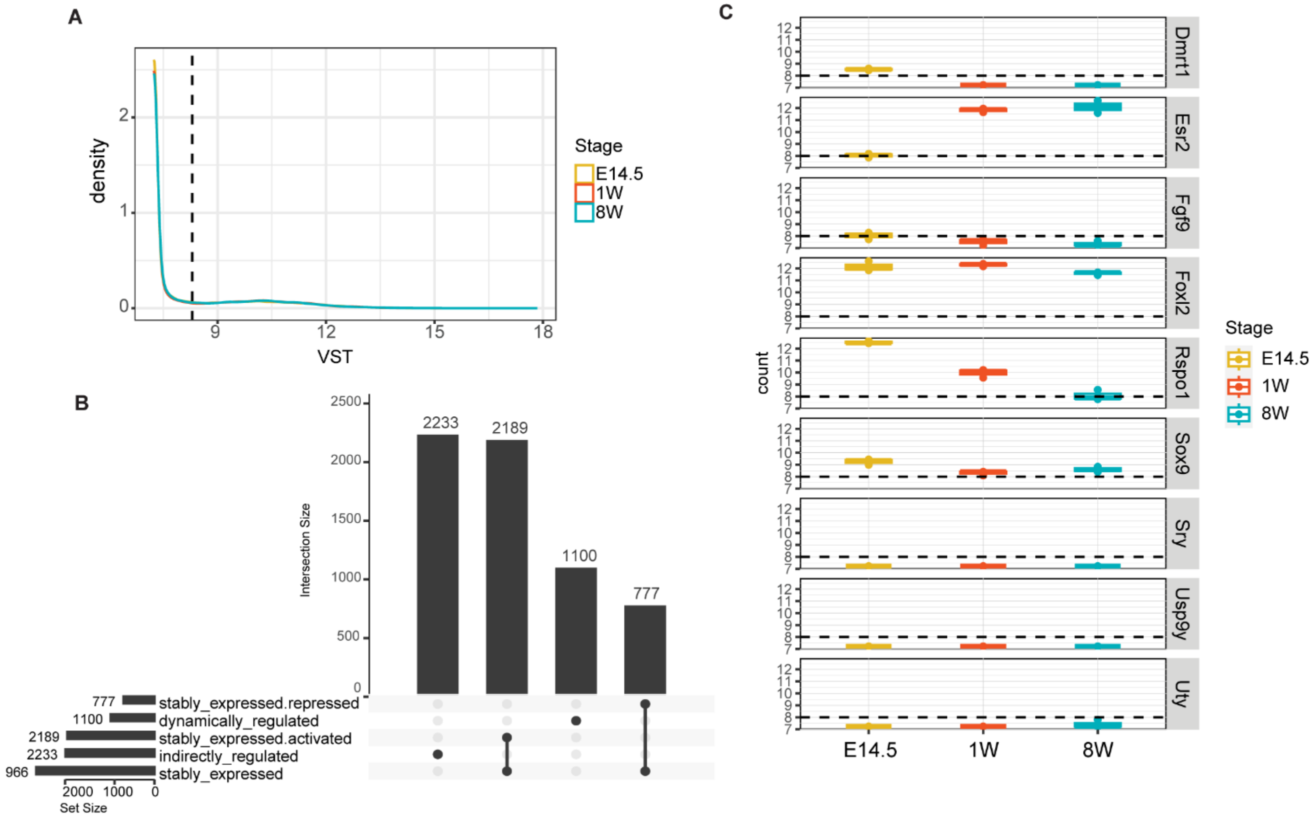

Fig. S12. Integration of FOXL2 ChIP-Seq data with RNA-Seq of *Foxl2*$^{EGFP/+}$ cells.
(**A**) Plot of gene density and VST cut-off =8.3 used to distinguish genes stably repressed from those stably expressed. (**B**) Bar plot depicting the number of genes either stably repressed or activated and bound by FOXL2. (**C**) Example of gene expression changes used to choose the VST cut-off. The male marker *Fgf9* was used as reference for the cut-off as known to not be expressed in the ovary.

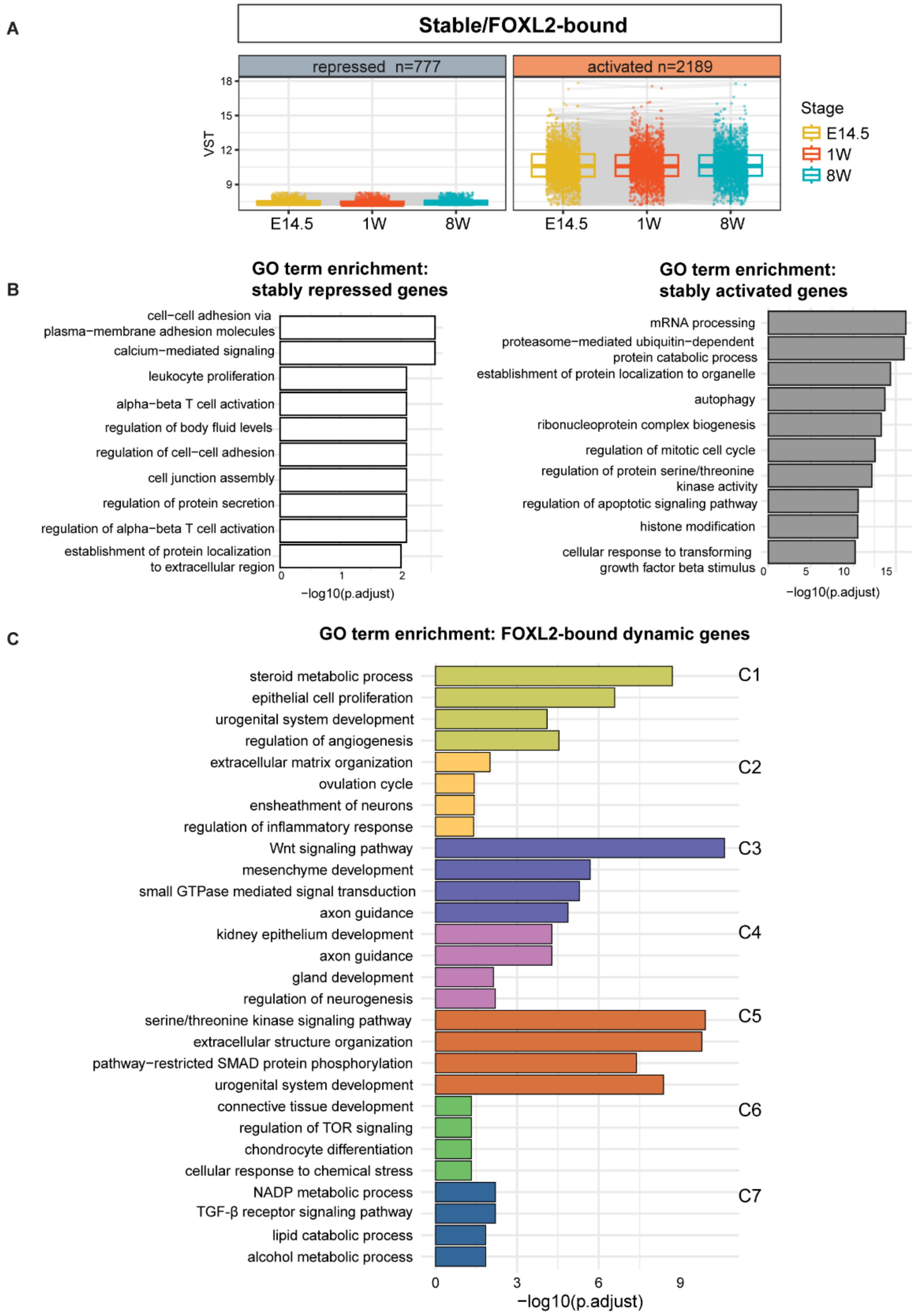

Fig. S13. Integrative approach combining RNA-Seq and ChIP-Seq to refine the gene regulatory networks controlled by FOXL2.

(**A**) Plot of variance stabilised (VST) gene expression values as detected by RNA-Seq on *Foxl2* $^{EGFP/+}$ sorted ovarian cells (n=3). (**B**) GO Biological processes enrichment analysis of stably repressed (left), and stably activated (right) genes. (**C**) GO enrichment analysis of the 1100 genes bound by FOXL2 and differentially expressed across the timecourse. Top four representative pathways are depicted.

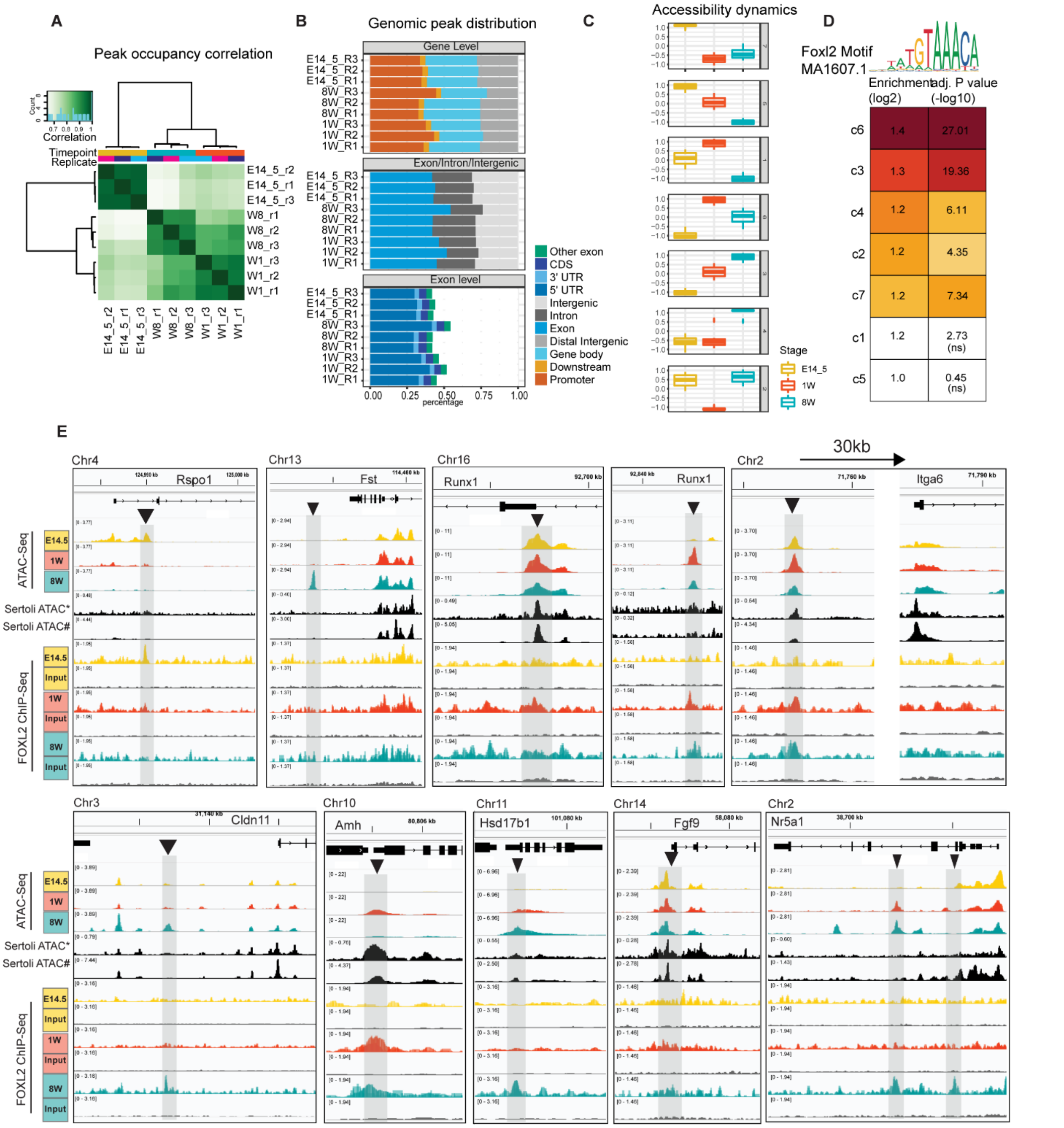

Fig.S14. ATAC-Seq analysis of chromatin accessibility of *Foxl2*^{EGFP} positive cells collected across ovarian development. (**A**) Poisson metric of peak occupancy correlation between timepoints. (**B**) Genomic annotation of consensus peaks. (**C**) Boxplot of z-scores representing overall trends of chromatin opening dynamics within each cluster derived from the hierarchical clustering of normalised abundancies of peaks. (**D**) Heatmap of enrichment scores for the FOXL2 canonical motif, ranked on enrichment score and filtered by -log10pval. Cut-off: -log10pval > 4 (i.e. pval<0.0001). (**E**) IGV snapshots representative of putative enhancers (grey boxes) identified within granulosa Sertoli cell marker genes, as assessed by ATAC-Seq and FOXL2 ChIP-SICAP. Black tracks show E13.5 Sertoli ATAC-Seq data re-analysed from *(118)* , and P7 Sertoli ATAC-Seq from (*50*).

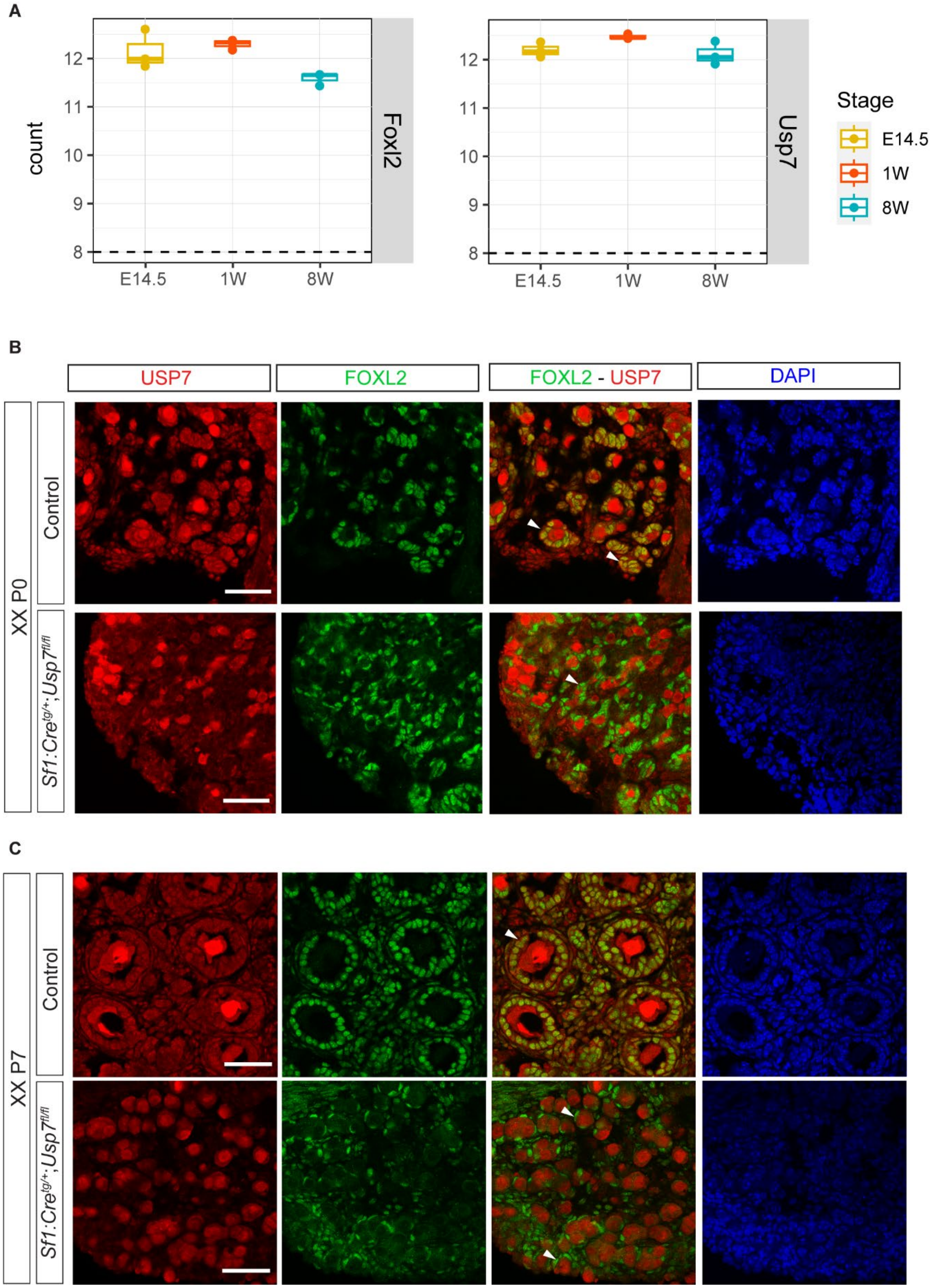

Fig.S15. Characterisation of *Usp7* mutants. (**A**) RNA-Seq expression values of FOXL2 and USP7 in developing ovaries. (**B**) Immunofluorescence analysis of FOXL2 and USP7 colocalisation in the somatic cell component of P0 and P7 (**C**) ovary. Scale bar = 50μM.

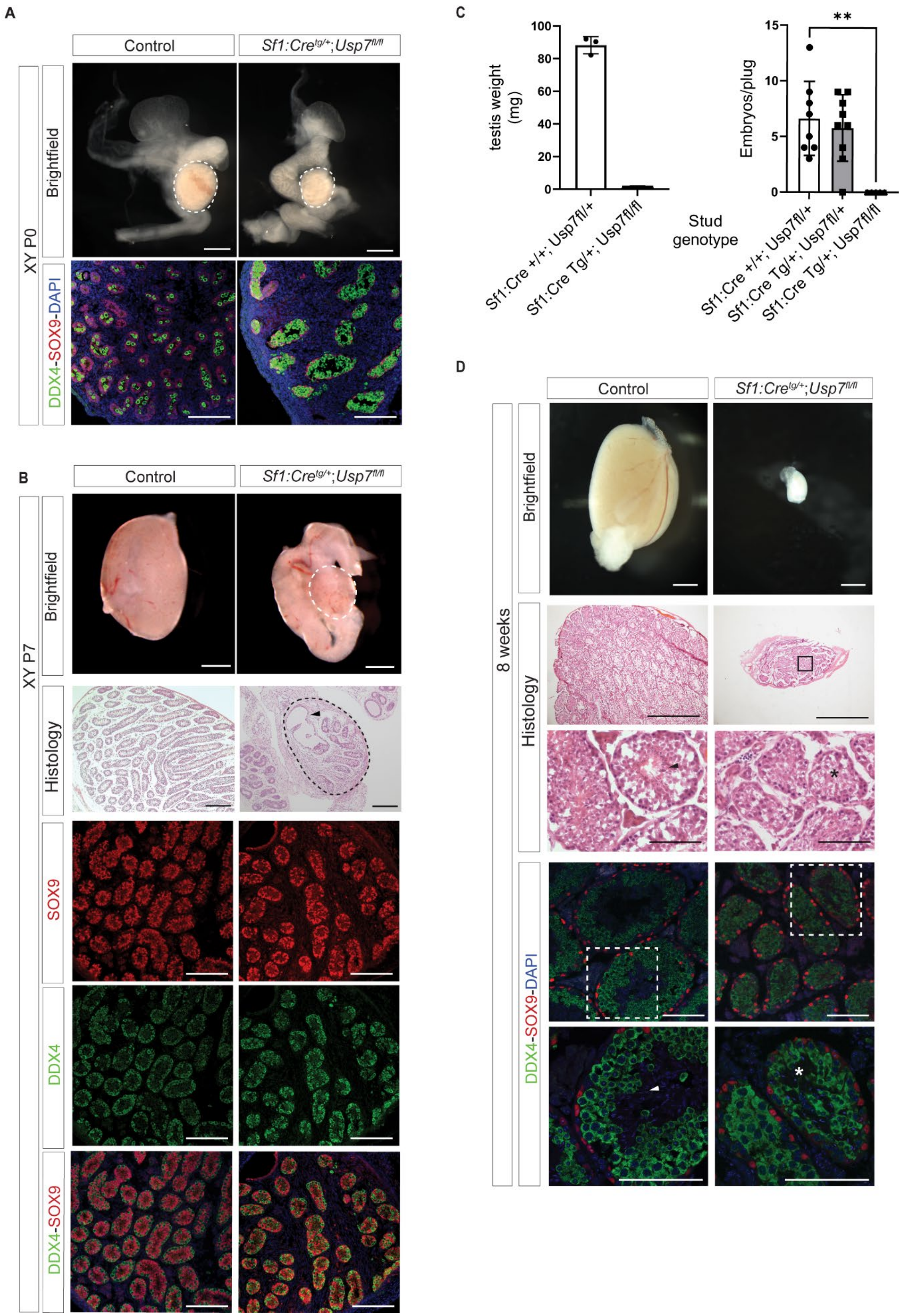

Fig. S16. Loss of *Usp7* in Sertoli cells leads to hypogonadisms and infertility in XY mice.

Characterisation of the Usp7 conditional knock-out phenotype in mouse XY testes. (**A**) Brightfield images showing gross morphology of control (*Usp7*$^{fl/fl}$*;Sf1:Cre*$^{+/+}$) and mutant testes (*Usp7*$^{fl/fl}$*;Sf1:Cre*$^{tg/+}$b) collected at birth (P0). Scale bars = 0.5 mm in all unless otherwise specified. Immunofluorescence analysis of germ cell marker DDX4 and Sertoli cell marker SOX9. (**B**) Brightfield images showing gross morphology of control and mutant testes collected 1 week postnatally (P7). Hematoxylin and Eosin (H&E) staining of section from control and mutant collected at P7 shows gonadal dysgenesis in mutant testes. Scale bar=200µm. Immunofluorescence analysis of DDX4 and SOX9 at P7. (**C**) Left: average testis weights of adult mice in mg. Data shown are mean and standard deviation from n=3 adult mice of each genotype. Right: number of born embryos per plug for male studs of the genotyped indicated and mated with control females. Data are mean and standard deviation from n=3 *Usp7*$^{fl/fl}$*;Sf1:Cre*$^{+/+}$ studs (8 plugs in total, control), from n=3 *Usp7*$^{fl/+}$*;Sf1:Cre*$^{tg/+}$ studs (9 plugs in total, heterozygous mutant), and from n=3 *Usp7*$^{fl/fl}$*;Sf1:Cre*$^{tg/+}$ studs (5 plugs in total, homozygous mutant). Asterisks denote p value<0.05, one-way Anova test. (**D**) Brightfield images showing gross morphology of control and mutant 8 weeks old testes. Scale bar= 1mm. H&E staining of sections from control and mutant testes. Arrowhead points to elongated spermatids in the lumen of the seminiferous tubule. Asterisk denotes absence of these in mutants. Scale bar=100µm. Immunofluorescence analysis of SOX9 in control and mutant testis sections indicates abnormal development and organisation of Sertoli cells (SOX9, red) and impaired spermatogenesis (DDX4, germ cells). Scale bar=500µm. All images are representative of at least 3 biological replicates.

Table S1.

Table of primers used to genotype the *Foxl2*^{EGFP} reporter strain.

Primer/Probe	Sequence
Insert F	aggacgacggcaactacaag (within EGFP sequence)
Insert R	gtccatgccgagagtgatcc (within EGFP sequence)
LR-F	gagaagagagtgagagccgc (from sequence upstream of 5' homology arm)
LR-R	gggtatctttgccggtgtga (from sequence downstream of 3' homology arm)
eGFP ddPCR F	agcagaagaacggcatca
eGFP ddPCR R	gggtgttctgctggtagtg
eGFP ddPCR Probe	caagatccgccacaacatcgagga
ONT-F1	tgtccattcgtgactgggac (from sequence upstream of 5' homology arm)
ONT-R1	gccgtcgtccttgaagaaga (within EGFP sequence)
ONT-F2	aggacgacggcaactacaag (within EGFP sequence)
ONT-R2	gatgaagtgaggggtgcaca (from sequence downstream of 3' homology arm)

Other supplementary data

Movie S1. HREM 3D rendition of adult reproductive tract from *Usp7*$^{fl/fl}$*;Sf1:Cre*$^{tg/+}$ XX mice.

Data S1: FOXL2 ChIP-SICAP analysis of genomic targets, GO of clusters and differential binding analysis.

Data S2: Comparison of FOXL2 ChIP-SICAP with other datasets from (*49*), (*50*) and (*29*).

Data S3: FOXL2 ChIP-SICAP, proteomics dataset of FOXL2 interactors.

Data S4: RNA-Seq of *Foxl2*$^{EGFP/+}$ cells isolated throughout ovarian development.

Data S5: Data integration of ChIP-Seq, ATAC and RNA-Seq and gene-level dynamics.

Data S6: ATAC-Seq normalised peak counts, differential binding analysis**,** clustering analysis.
